# Modelling the Spread of HIV Immune Escape Mutants in a Vaccinated Population

**DOI:** 10.1371/journal.pcbi.1002289

**Published:** 2011-12-01

**Authors:** Helen R. Fryer, Angela R. McLean

**Affiliations:** The Institute for Emerging Infections, The Oxford Martin School, Department of Zoology, Oxford University, Oxford, United Kingdom; Utrecht University, Netherlands

## Abstract

Because cytotoxic T-lymphocytes (CTLs) have been shown to play a role in controlling human immunodeficiency virus (HIV) infection and because CTL-based simian immunodeficiency virus (SIV) vaccines have proved effective in non-human primates, one goal of HIV vaccine design is to elicit effective CTL responses in humans. Such a vaccine could improve viral control in patients who later become infected, thereby reducing onwards transmission and enhancing life expectancy in the absence of treatment. The ability of HIV to evolve mutations that evade CTLs and the ability of these ‘escape mutants’ to spread amongst the population poses a challenge to the development of an effective and robust vaccine. We present a mathematical model of within-host evolution and between-host transmission of CTL escape mutants amongst a population receiving a vaccine that elicits CTL responses to multiple epitopes. Within-host evolution at each epitope is represented by the outgrowth of escape mutants in hosts who restrict the epitope and their reversion in hosts who do not restrict the epitope. We use this model to investigate how the evolution and spread of escape mutants could affect the impact of a vaccine. We show that in the absence of escape, such a vaccine could markedly reduce the prevalence of both infection and disease in the population. However the impact of such a vaccine could be significantly abated by CTL escape mutants, especially if their selection in hosts who restrict the epitope is rapid and their reversion in hosts who do not restrict the epitope is slow. We also use the model to address whether a vaccine should span a broad or narrow range of CTL epitopes and target epitopes restricted by rare or common HLA types. We discuss the implications and limitations of our findings.

## Author Summary

The evolution and spread of HIV strains that evade the immune response poses a major challenge to the development of an effective and robust HIV vaccine. We present a new mathematical tool that we use to dissect the drivers of the spread of these ‘immune escape mutants’ in a vaccinated population. Our study focuses on a vaccine that can reduce infectiousness and enhance longevity but does not provide sterilizing immunity. We show that in the absence of escape such a vaccine could reduce the prevalence of both infection and disease in the population. However, vaccine impact could be significantly abated by immune escape mutants, especially if they emerge rapidly and revert very slowly after transmission to hosts in whom the original selection pressure is absent. We also discuss the effect that vaccine breadth and the frequency with which different epitopes are targeted have upon vaccine impact.

## Introduction

The development of a HIV vaccine is one of the key global health priorities of our time. Early vaccine candidates aimed to elicit antibodies but conclusively failed in their goal of providing sterilising immunity [1]. It is now clear that many challenges exist in engendering effective antibodies [2], [3]. It is also now apparent that immune cells called cytotoxic t-lymphocytes (CTLs) play an important role in controlling viral replication during natural HIV infection [4]–[8] and that eliciting these responses – alone or alongside antibodies – may be key to vaccination success.

Because CTLs target infected cells rather than free virus a vaccine that induces only CTL responses would be unlikely to provide sterilizing immunity; instead a realistic aim is for such a vaccine is to contain the virus at levels that are low enough to prevent onwards transmission and slow the onset of AIDS. Successful trials of CTL-based SIV and SHIV vaccines in non-human primates provide hope that this may one day be an achievable goal [9]–[13]. Notably, Hansen at al. recently demonstrated that CTLs were responsible for markedly reducing viral replication in macaques experimentally infected with a highly pathogenic strain of SIV [12]. However, the success that has been had in macaques has not yet been translated to humans. The only large scale human trial of an HIV vaccine specifically designed to elicit CTLs was not successful [14]. Not only did the MRKAd5 vaccine fail (as anticipated) to prevent infection, it also failed in its main objective of reducing viral replication in those who became infected.

The only positive news from large scale human vaccine trials has recently come from a Thai study in which high risk individuals were inoculated with a combination of two immunogens – one intended to induce antibodies, the other to induce CTLs [15]. This combined approach was reported to reduce infection probability by 31% over a 3 years study period. The result, however, was borderline significant, appeared to wane over time and was scrutinised over the inclusion of certain individuals in the statistics [16]. Furthermore, the immunological analysis is pending, thus a number of questions remain about this study including the nature of any protective immune responses, the reasons for their demise and the ability to reproduce and improve upon this result in future trials. The precise makeup of an effective vaccine therefore remains unclear and current trials continue to explore the capacity of both the humoral and cellular arms of the immune system (www.iavi.org).

One of the challenges of vaccine research lies in HIV's ability to evolve mutant strains that evade immune responses [6], [7], [17]. These ‘escape mutants’ pose a particular type of problem to the development of a CTL-based vaccine that, as prototyped in macaques [12], could suppress viral replication in infected hosts, but not prevent infection. Under such a vaccination scenario, CTL escape mutants could be generated within vaccinated hosts [18], [19], transmitted between hosts [20], [21] and accumulate at the population level [22]–[24]. A better understanding is needed of how CTL escape mutants will evolve in a vaccinated population, what effect they will have on the impact of a vaccine, and how vaccines should be designed to reduce their impact.

Several factors, in addition to the rate at which mutants are selected, are likely to influence the evolution of CTL escape mutants and the impact of a CTL-based vaccine at the population level. One consideration is the huge level of diversity amongst the population in the genes that encode the human leukocyte antigen (HLA). HLA class I genes determine the epitopes (antigenic sections of viral protein) to which each individual can make CTL responses. This means that different hosts make responses to (or ‘restrict’) different epitopes. Whilst vaccination will not be able to change the epitopes that each individual can restrict, it is hoped that it will be able to improve the responses to those epitopes they do restrict, and make them come into play earlier on during infection. As different hosts restrict different epitopes they also drive the evolution of different escape mutants and a mutation that is advantageous to the virus in one individual will not necessarily be advantageous when transmitted to another. Furthermore, because mutations can impose a fitness cost on the virus [25], [26] reversion of escape mutations can occur following transmission to a new host [27], [28]. The spread of escape mutants at the population level is therefore also likely to be influenced by reversion rates and the frequencies of different HLA types in the population.

Another factor that could affect the impact of a vaccine is the breadth of the CTL response that the vaccine elicits. HLA diversity means that any vaccine capable of providing protection throughout a population would have to encode protein that spans a range of different CTL epitopes restricted by different HLA-alleles. A broader range of epitopes would not only satisfy different HLA-restrictions, but might also be more robust in the event of escape.

In this study we investigate how CTL escape mutations are likely to evolve in a vaccinated population and how CTL escape could affect the impact of an HIV vaccine. Specifically, we ask the following four questions:

- How would a CTL-inducing vaccine affect the progression of an HIV-epidemic?
- How would selection, reversion and transmission of CTL escape mutants affect the impact of a vaccine?
- Should a vaccine span a broad or narrow range of CTL epitopes?
- Should a vaccine target epitopes restricted by rare or common HLA alleles?

To address these questions we have created a simple mathematical model that describes a population receiving a vaccine which induces CTL responses to multiple epitopes. Crucially, the model includes within-host evolution and between-host transmission of viral variants that have escaped from CTL responses. It is a natural extension of a model that we previously developed to investigate the evolution and spread of CTL escape mutants in an unvaccinated population [22].

## Results

### Modelling the spread of escape mutants in a vaccinated population

The model that we have developed represents the dynamics of escape in a population that receives a vaccine that induces CTL responses. It is assumed that the vaccine can confer better viral control, resulting in an enhanced life expectancy and a reduction of infectiousness. For simplicity we focus this study on modelling a wholly CTL-based vaccine that does not provide sterilizing immunity, i.e. does not prevent infection in vaccinated hosts. However, there is flexibility in the model to consider a vaccine that also provides a degree of sterilizing immunity and we comment on such model results later. The vaccine induces CTL responses to multiple epitopes and to represent host heterogeneity in HLA types, each epitope can be recognised by only a fraction of the population. To represent viral diversity there are different strains of the virus such that at each epitope the virus can take the wildtype or escape mutant form. For simplicity, there are no mixed infections, or more precisely, each host can only be infectious with one strain at any given time. A strength of this model is that it captures events whilst viruses evolve within individuals and tracks the spread of variants as viruses are transmitted between individuals. An individual who is infected with a virus that is the wild-type form at an epitope that their HLA can restrict can mount an effective CTL response to that epitope. Such an individual can drive the evolution of an escape mutant at that epitope and can therefore switch to becoming infected with a strain that has the escape mutant at that epitope. A host who is HLA mismatched for a particular epitope is unable to mount a CTL response to that epitope, irrespective of vaccination and the mutations it bears at that epitope, thus their infecting virus can revert from an escape mutant to the wild-type strain at that epitope. The rate of reversion is the same in both vaccinated and unvaccinated hosts.

**Figure 1:**
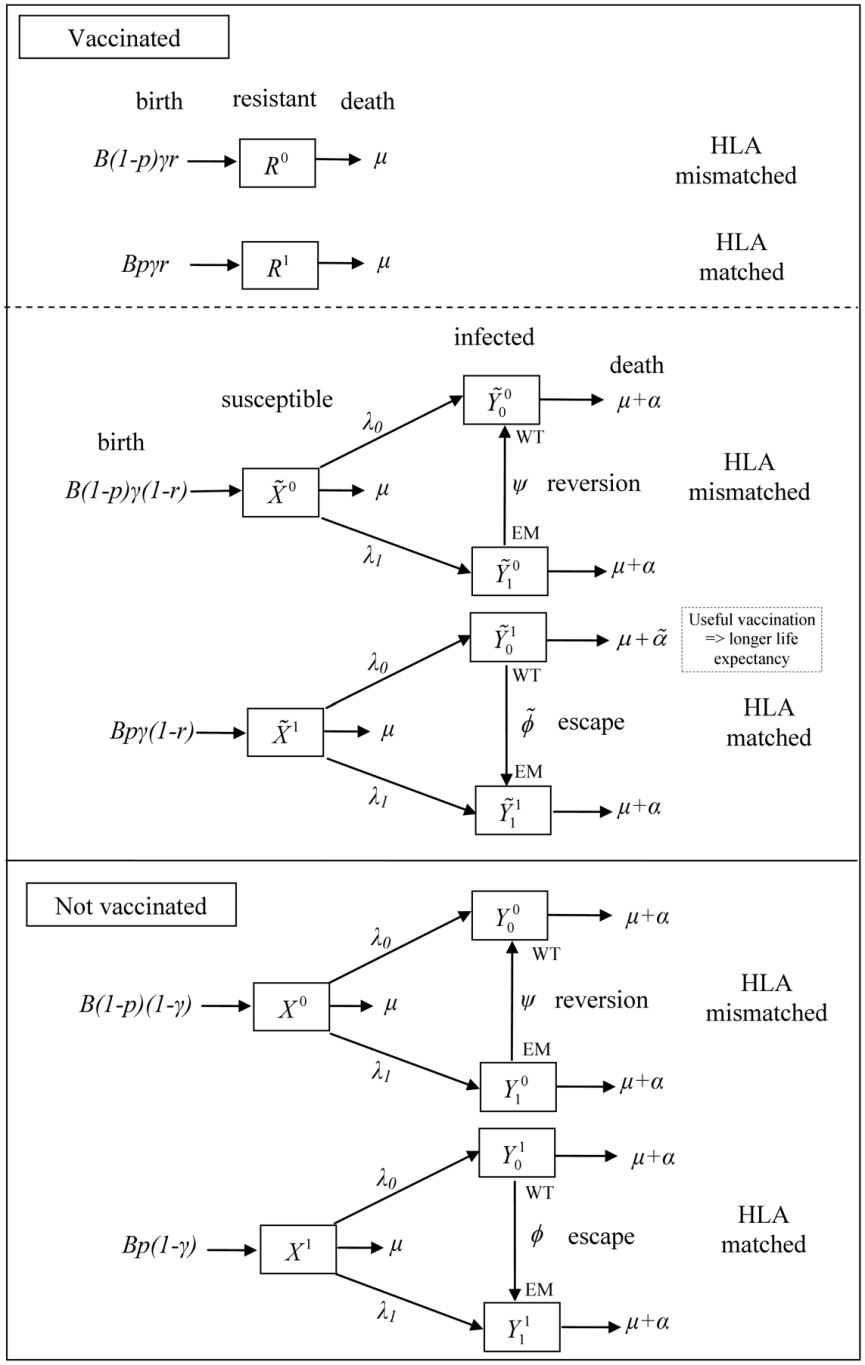
A schematic form of the single epitope version (n = 1) of the mathematical model of within-host evolution and between-host transmission of escape mutants in a vaccinated population. In this model between-host transmission is modelled using a standard mathematical description of the frequency-dependent transmission of an infectious disease from which there is no recovery [29]. However, it includes extra stages of compartmentalisation representing whether the hosts are 1) HLA matched or mismatched for the epitope; 2) susceptible vaccinated, resistant vaccinated or unvaccinated and 3) whether the infected hosts have the wildtpe (WT) or the escape mutant (EM) strain. There are two additional processes: escape in HLA matched hosts infected with the wildtype strain and reversion in HLA mismatched hosts infected with the mutant strain. Escape occurs at rate 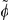 in vaccinated hosts and rate 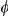 in unvaccinated hosts. Reversion occurs at rate 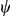 in both host types. Infected vaccinated hosts who are HLA matched for the vaccine epitope and who do not have an escape mutant at that epitope have a longer life expectancy compared to other infected hosts (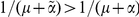). Such individuals are also less infectious so make a smaller per capita contribution to the force of infection (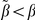). Thus the force of infection for wildtype virus is defined as 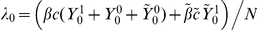 and for escape mutant virus is defined as 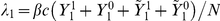.

In this model the presence of CTL escape mutations affects the effectiveness of the vaccine. The enhancement to life expectancy and reduction in infectiousness conferred by the vaccine only applies to hosts who are infected with virus that is wildtype at at least one of the vaccine epitopes for which they are HLA-matched. Each epitope is assumed to act independently. Thus, each response is either beneficial or neutral to viral control, the impact of a vaccine is independent of *which* epitopes there are responses to and escape rates at each epitope are independent of both the presence of escape mutants at other epitopes and the contribution that other epitopes make to viral control. Between-host transmission is modelled using a standard mathematical description of the frequency-dependent transmission of an infectious disease from which there is no recovery [29]. Every infected host is infectious with the viral type they carry, so that the different viral types are transmitted between individuals at rates driven by the proportion of the total population infected with each. A mathematical description of the model and the ordinary differential equations prescribing the model (Equation 1), are provided in the methods section. A description of model variables (Table 1), model parameters (Table 2) and additional terms used to enable the model equations to be written in a simple and general form (Table 3) are also provided. Finally, a schematic diagram of the model for the special case where the vaccine contains only a single epitope is shown in Figure 1.

**Table 1:** A description of variables used to describe our model.

Symbol	Description
*t*	Time
*i*	Epitope number (from 1 to *n*)
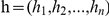	Host type, indicating which of the *n* epitopes the host restricts: 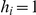 if the host restricts epitope *i* and 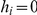 if it does not.
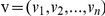	Virus type, indicating the epitopes at which the virus carries escape mutants: 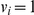 if the virus is mutant at epitope *i* and 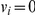 if it is wildtype.
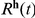	The number of vaccinated resistant hosts of type **h** at time *t*
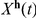	The number of unvaccinated susceptible hosts of type **h** at time *t*
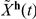	The number of vaccinated susceptible hosts of type **h** at time *t*
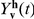	The number of unvaccinated type **h** hosts infected with virus type **v** at time *t*.
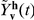	The number of vaccinated type **h** hosts infected with virus type **v** at time *t*.
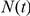	The total number of hosts in the population at time *t*.
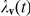	Force of infection from infected hosts with virus type **v** at time *t*.

**Table 2:** Definitions of the model parameters and a description of the parameter values used in our simulations.

Symbol	Description	Value used in simulation	Interpretation of parameter
*μ*	Population death rate	1/50 years^−1^	Uninfected hosts have an average life expectancy of 50 years. This estimate is based upon a population in Southern Africa [36].
B	Population birth rate	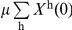	The population size is constant in the absence of infection.
*γ*	Proportion of newborns who receive the vaccine at birth.	0 for the first 50 years of the epidemic and 1 beyond year 50.	No hosts receive the vaccine between years 0 and 50. Beyond year 50 all newborns receive the vaccine. Note that in addition we have assumed that all other hosts receive the vaccine at year 50.
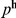	Proportion of hosts in the population with host type **h**	In Figure 7 different proportions are compared: 0.1^k^0.9^n-k^, 0.3^k^0.7^n-k^ and 0.5^k^0.5^n-k^, where 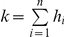 is the number of epitopes for which the host is HLA matched. In the remaining figures the proportion is 0.2^k^0.8^n-k^	Recognition of each epitope is assumed to be independent. In Figure 7 different percentages of the population recognising each epitope are considered: 10%, 30% and 50%. In the remaining figures 20% of the population recognise each epitope.
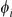	Rate of escape at epitope *i* in unvaccinated HLA matched hosts.	In Figure 3 different escape rates (the same rate at each epitope) are compared: 1/3 years^−1^, 1/30 years^−1^ and 0 years^−1^. In Figure 5 rates ranging from 0 years^−1^ to 1/1000 years^−1^ are considered. In the remaining figures the rate at each epitope is 1/8 years^−1^ [22].	The reciprocal of the rate of escape is equal to the average time between infection and escape.
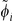	Rate of escape at epitope *i* in vaccinated HLA matched hosts.	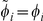	At each epitope escape occurs at the same rate in vaccinated and unvaccinated hosts.
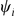	Rate of reversion at epitope *i* in HLA mismatched hosts.	In Figure 4 different reversion rates (the same at each epitope) are compared: 1/3 years^−1^, 1/30 years^−1^ and 0 years^−1^. In Figure 5 rates ranging from 0 years^−1^ to 1/1000 years^−1^ are considered. In the remaining figures the rate at each epitope is 1/36 years^−1^ [22].	The reciprocal of the rate of reversion is equal to the average time between infection and reversion.
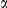	Disease-related death rate of unvaccinated or unsuccessfully vaccinated hosts	(1/10-µ) years^−1^	The average life expectancy in the absence of vaccine-induced protection (1/(µ+α)) is 10 years [37]. This estimate assumes that infected unvaccinated hosts do not receive treatment.
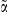	Disease-related death rate of successfully vaccinated hosts.	0 years^−1^	Successfully vaccinated hosts have the same life expectancy as uninfected hosts.
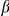	Transmission rate per partnership with an unvaccinated or unsuccessfully vaccinated host	0.2/c	In an unvaccinated population this yields a basic reproduction number of 2 [33].
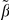	Transmission rate per partnership with a successfully vaccinated hosts.	In Figure 2 different values are considered: 0.008/c and 0.1/c. In the remaining figures: 0.008/c	In Figure 2 successfully vaccinated hosts are 25 times or 2 times less infectious compared to unvaccinated or unsuccessfully vaccinated hosts. In the remaining figures they are 25 times less infectious.
*c*	Rate of partner exchange	See above	See above.
*r*	Fraction of vaccinated hosts resistant to infection	0	No hosts are resistant to infection
*n*	Number of epitopes in the vaccine	In Figure 6 different number of epitopes are considered: 1, 3 and 5. In the remaining simulations there are 5 epitopes.	This is the number of epitopes that the vaccine is capable of inducing CTL responses to.

**Table 3:** Additional terms referred to in the text.

Symbol	Description
*F*	The set of pairs 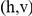 which have 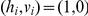 for at least one epitope *i*, then a vaccinated host of type **h** who has virus type **v**, such that 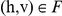 , is successfully vaccinated, i.e. they restrict at least one epitope they are vaccinated against and that epitope has no escape mutations. By contrast, a vaccinated host with 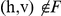 is unsuccessfully vaccinated and has the same properties as an unvaccinated infected host.
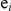	The unit vector of length *n* that has *i*th coordinate equal to 1, e.g. 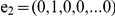
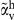	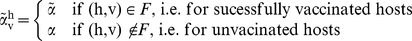
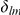	The Kronecker delta: 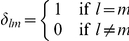
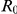	The basic reproductive number. The expected number of secondary cases caused by one primary case in a wholly susceptible population. In this model framework 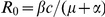 .
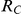	The control reproductive number. The expected number of secondary cases caused by one primary case in a wholly uninfected population under a control strategy.
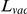	Lifetime transmission potential of a successfully vaccinated host. In this model framework 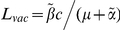 .
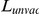	Lifetime transmission potential of an unvaccinated or unsuccessfully vaccinated host. 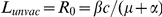 .
**K**	The next generation matrix

We have used the model to simulate the application of a vaccine to all hosts in a population 50 years into an epidemic and to all newborns thereafter (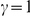). The vaccine is assumed to be effective only in hosts who are not infected at the time of vaccination. As the main purpose of this study is to explore the underlying factors that drive the effectiveness of vaccination we have deliberately kept application of this model as simple as possible. Because of computational limitations, one of the main simplifications that we make in our simulations is that the vaccine can elicits responses to only five CTL epitopes 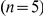. Given the huge variability in HLA types amongst the population, each epitope has the potential to be recognised by only a small fraction of the population, thus any vaccine will have to elicit a much broader response than modelled here if it is to provide robust protection to the majority of individuals. Because it is not computationally feasible to increase the modelled vaccine breadth further, we have attempted to maximise vaccine coverage by assuming that all hosts receive the vaccine (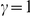) and that a large fraction (20%) of hosts are HLA matched for each epitope. In reality, HLA prevalences below 10% [30] and a vaccine coverage below 80% would be more typical [31]. To restrict unrealistic rapid spread of escape at each epitope, we did not increase the restricting HLA prevalence at each epitope any further. Recognition of each epitope is assumed to be independent (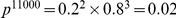), meaning that the proportion of hosts restricting each number of epitopes (0 to 5) is described by a binomial (5,0.2) distribution: 33% recognise no epitopes, 41% recognise one epitope, 20% recognise two epitopes and 6% recognise three or more epitopes. The median number of epitopes recognised is therefore only one, much lower than expected in reality (at least seven epitopes [32]), meaning that our simulations likely provide conservative estimates of the impact of vaccination.

Whereas we explicitly model only a limited number of vaccine epitopes, an implicit assumption of this model is that all hosts make natural responses to (perhaps many) more epitopes than just those included in the vaccine. Thus, whilst we assume that vaccine-driven responses to unmutated epitopes included in the vaccine can enhance life expectancy, natural responses to unmutated epitopes included in the vaccine are assumed to have no impact upon life expectancy. In reality, escape mutants may affect the life expectancy of unvaccinated hosts; however, we make this assumption because we are most interested in the effect of escape mutants upon vaccine impact, rather than their impact upon life expectancy in natural infection.

Another simplification of our approach pertains to the epidemic dynamics (i.e. infection prevalence over time) borne out by our simple model. We loosely base our simulations upon an epidemic in a high risk southern African population assuming that the basic reproductive number (R_0_) in the absence of vaccination is equal to 2, as recently estimated from a population in Zambia [33]. This epidemiological metric defines the average number of secondary cases caused by one primary case in a wholly susceptible population. However, even using an R_0_ tailored to Zambia does not fully reproduce the epidemics dynamics observed in that population, where infection prevalence appears to have stabilised at around 15% (lower than the equilibrium prevalence of 50% achieved with an R_0_ of 2), despite a continual increase in infected numbers coupled with marked population growth. Various factors could contribute to this discrepancy including heterogeneity of risk across individuals. Nevertheless, we use this simplified representation of transmission for reasons of transparency, analytical tractability and consistency with other modelling approaches [22], [29]. Accordingly, the broad dynamics indicated by this study should be regarded as more meaningful than the precise numerical findings. We leave more detailed tailoring of vaccine models to specific populations for future studies.

### How would a CTL-inducing vaccine affect the progression of an HIV epidemic?

Using this model we have considered the impact that vaccination would have on the proportion of hosts in the population infected with HIV (HIV prevalence) and on the proportion of hosts with uncontrolled HIV infections. Here ‘uncontrolled infections’ represent all infections except those in which the host is vaccinated and escape mutations have not rendered the vaccine useless. Thus, only hosts with uncontrolled HIV have the potential to progress to AIDS. Vaccination is assumed to be successful (i.e. control infection) in a particular host when the vaccine contains at least one epitope which that host restricts and in which that host's infecting virus does not have escape. This is illustrated in Text S1. Successful vaccination confers a longer life expectancy and a reduction in infectiousness compared to other infected individuals.

To model the spread of escape mutants amongst the population, we have assumed that escape takes an average of 8 years post infection at each epitope in both vaccinated (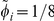
years^−1^ for *i* = 1∶5) and unvaccinated (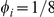
years^−1^ for *i* = 1∶5) HLA matched hosts [22]. At each epitope, reversion in HLA mismatched hosts is assumed to take an average of 36 years post infection (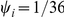 years^−1^ for *i* = 1∶5) [22]. We further explore these parameter choices in the next section.

**Figure 2:**
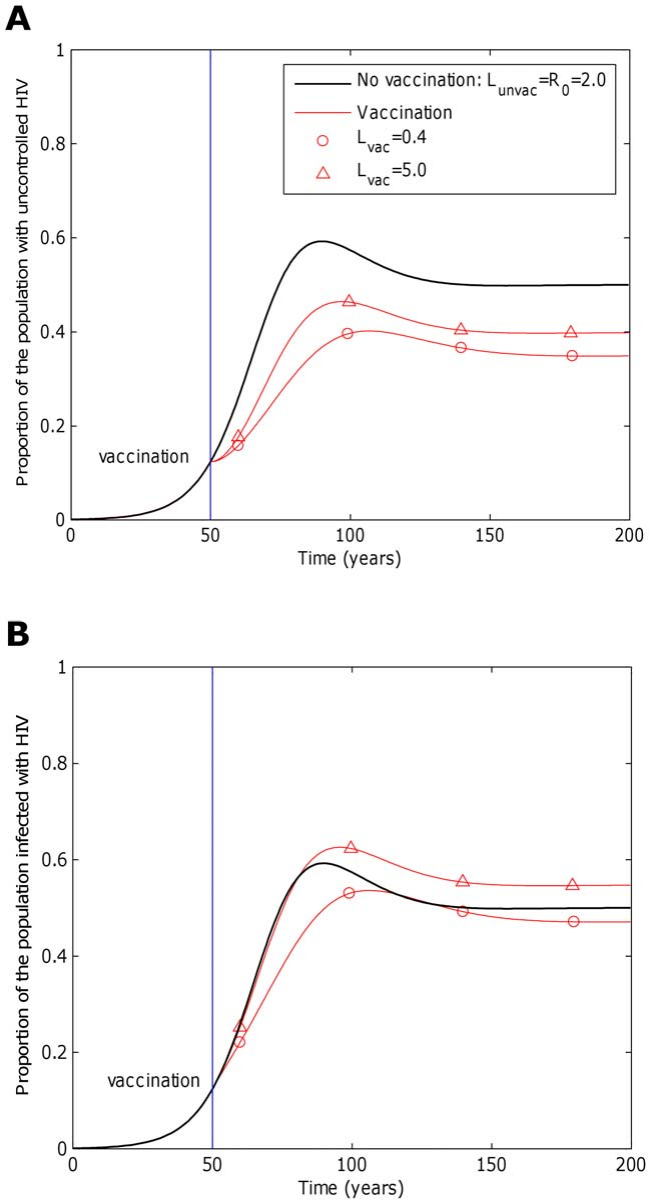
The impact of a CTL-based vaccine is dependent upon the extent to which it affects transmission potential. This figure considers the impact that a five-epitope vaccine could have on an epidemic. Two measurements are considered: A) the proportion of hosts in the population with uncontrolled infections and B) the proportion of hosts who are infected. In each panel two vaccines with different average transmission potentials are explored and compared to the scenario when the vaccine is absent (black line). Our simulations are based upon an epidemic in a sub-saharan community, thus we assume a basic reproductive number of 2 in the absence of vaccination (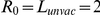,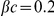,
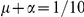
years^−1^). One vaccine (circles, A and B) considerably reduces transmission probability by a factor of 25 (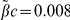), but restores life expectancy to its normal value (50 years; 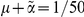 years^−1^). This vaccine reduces transmission potential by a factor of 5 (from 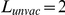 to 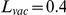). The second example vaccine (triangles, A and B) causes a modest two fold reduction in transmission probability (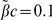) and restores life expectancy to the its uninfected value (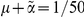). Thus, this vaccine more than doubles the transmission potential (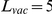). In both cases the vaccine is administered to all unvaccinated hosts 50 years into the epidemic and to all newborns (*γ* = 1) thereafter, but provides no level of sterilizing immunity (*r* = 0). This figure shows that a vaccine that reduces the transmission potential (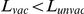) – i.e. suppresses infectiousness by a greater factor than it increases life expectancy – would reduce both the proportion of hosts with uncontrolled HIV (circles, A) and the proportion of hosts infected with HIV (circles, B). A vaccine that reduces transmission potential (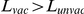) would also decrease the proportion of hosts with uncontrolled HIV (triangles, A), but would marginally increase the proportion of hosts infected with HIV (triangles, B). For these figures we assume that the vaccine elicits responses to five CTL epitopes. At each epitope escape in HLA matched hosts escape takes an average 8 years following infection (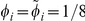 years^−1^ for *i* = 1∶5). Reversion in HLA mismatched hosts takes an average of 36 years at each epitope (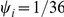 years^−1^ for *i* = 1∶5). We also assume that at the start of the epidemic 0.1% of the population are infected and that all of these hosts are infected with the wildytype strain.

Our model predicts that even a vaccine that does not prevent hosts from becoming infected could have an impact upon an HIV epidemic. The proportion of people with uncontrolled infections could be reduced by vaccination (Figure 2A) since many people who become infected would stem the normal progression of the infection towards disease, at least temporarily, because of the protection offered by the vaccine. The proportion of people infected with HIV could also be reduced (Figure 2B, circles) since hosts who are protected by a vaccine would be less infectious compared to unvaccinated hosts (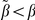). These basic dynamics are reproduced under different assumptions about the underlying epidemic, however the numerical results differ. This is demonstrated in Figure S1 by considering epidemics parameterised by different basic reproductive numbers.

Precisely whether or not a vaccine has a positive impact upon the proportion of the population infected, however, also depends upon the extent to which successful vaccination extends life expectancy and thus extends the period over which hosts can transmit. To demonstrate this, we introduce the concept of the lifetime transmission potential, the product of the transmission coefficient (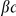) of the host and the average life expectancy of the host (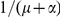). This value, which we denote *L*, differs depending upon whether the host is successfully vaccinated or unvaccinated – a term we use here to describe hosts who have either not received the vaccine or who are unsuccessfully vaccinated. For these two respective host types, we define the lifetime transmission potential as 
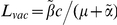
 and 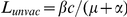. Note that 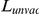 is identical to the basic reproductive number.

If successful vaccination leads to a greater factor reduction in infectiousness than factor increase in life expectancy 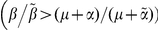, 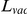 would be lower than 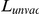 and vaccination would lead to a reduction in the proportion of people infected (Figure 2B, circles) [34],[35]. By contrast, if the factor reduction in infectiousness is less than the factor increase in life expectancy (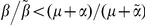), in the long term vaccination would increase the proportion of people infected (Figure 2B, triangles).

It is noteworthy that there exists an upper bound on the extent to which a CTL-based vaccine could increase infection prevalence in the population because the average life expectancy of successfully vaccinated hosts would not exceed that of uninfected hosts (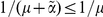) and successfully vaccinated hosts are unlikely to be more infectious than unvaccinated hosts (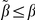). This means that 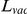 cannot exceed 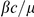 – i.e. the factor increase in 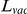 compared to that of 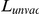 can be no more than 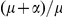. It follows that any vaccine that reduces infectiousness by a factor of at least 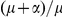 would reduce infection prevalence. As an example, in sub-Saharan Africa where the average life expectancy of uninfected hosts is approximately 50 years [36] and the average life expectancy of untreated infected hosts is approximately 10 years [37], a vaccine that reduces infectiousness by a factor of 5 (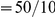) or more would be sure to reduce the prevalence of infection. A vaccine that does not fully restore life expectancy to pre-infection levels could reduce infection prevalence with a lower factor reduction in infectiousness.

Whilst it is not possible to precisely predict the way in which vaccination would affect the lifetime transmission potential, the way in which this metric varies across different individuals with natural infection provides us with some clues. It is now clear that plasma viral load is a predictor of both transmission probability [33], [38]–[42] and the life expectancy of untreated hosts [33], [43]–[45]. A review of these data presented in Fraser et al. [33] suggest that with each tenfold increase in viral load, transmission probability per contact increases approximately linearly and life expectancy decreases approximately linearly. The authors also explored the combined effect of viral load and life expectancy on lifetime transmission potential at different viral loads. Their analysis suggests that lifetime transmission potential is greatest at a viral load of 4.5 log_10_ copies per millilitre. This happens to equal the global median set point plasma viral load [44] implying that, averaged across a population, a vaccine that reduces the average viral load will be sure to reduce lifetime transmission potential and thus reduce both the prevalence of infection and uncontrolled infection in the population.

The precise reduction in lifetime transmission potential that can be expected from vaccination, however, remains unclear. At the lowest dose for which estimates were available (3 log_10_ copies per millilitre) Fraser et al. [33] predicted that lifetime transmission potential would be reduced by a factor of five compared to its maximum at 4.5 log_10_ copies/ml. It is hoped, however, that vaccination can reduce viral loads to lower values than this and ideally below the standard detection level of 50 copies/ml. This level of viral suppression has been observed in macaques successfully vaccinated with a CTL-based SIV vaccine [12] and elite controllers [46], [47] – individuals who successfully control natural HIV and exhibit normal life expectancies in the absence of drugs. Whilst data on the lifetime transmission potential of elite controllers is limited, data from a recent antiretroviral drug trial [48] suggest that treatment (which typically reduces viral loads below 50 copies/ml) can reduce the likelihood of transmission probability by at least a factor of 25 (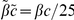). If successful vaccination could restore life expectancy to uninfected levels (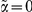), this would also result in a fivefold reduction in lifetime transmission potential. In the reminder of our simulations we have therefore used these parameters (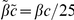 and 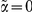 years^−1^), to assume a fivefold factor reduction in lifetime transmission potential. In Figure 2, this best estimate is represented by the circles. However, it is noteworthy that whilst the reductions presented here are rather modest, a vaccine that contains many more epitopes than we simulate could have markedly greater impact.

### How would selection, reversion and transmission of CTL escape mutants affect the impact of a vaccine?

Our model simulations show that CTL escape could have a considerable effect on the impact of a vaccine. Not only would it affect the individuals in whom the mutants are selected, but it could also affect the impact of the vaccine at the population level. Our model assumes that vaccinated hosts who lose viral control due to escape revert to normal infectiousness, hence transmit infection to others as though they had not been vaccinated. Furthermore they transmit viral strains that carry vaccine escape mutants, leading to an increase in the prevalence of escape mutants at the population level.

**Figure 3:**
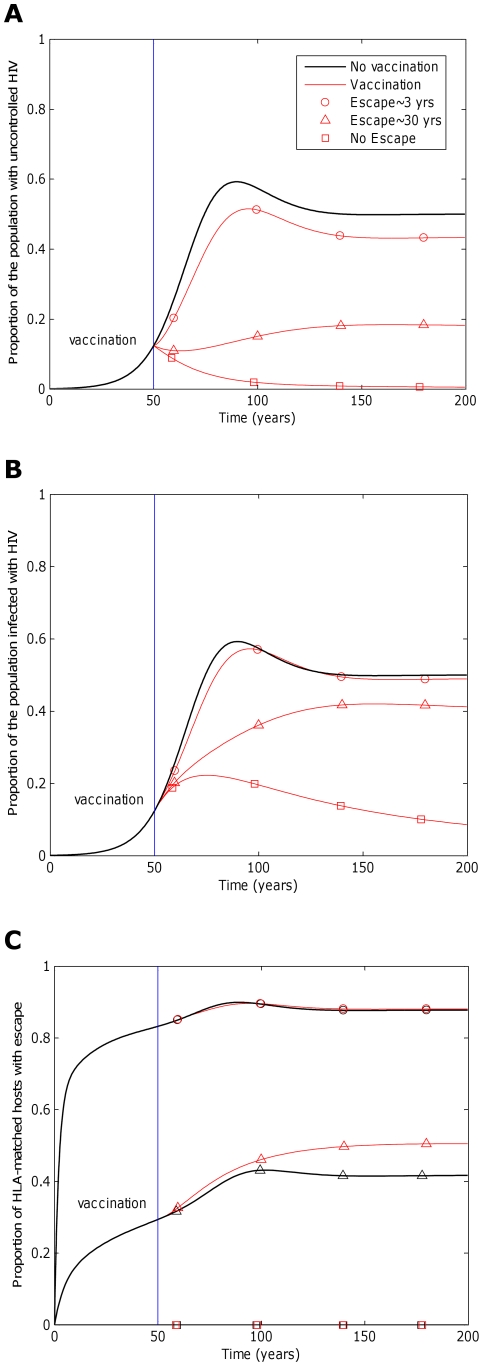
The impact of a vaccine will be greater if the rate at which immune escape mutants emerge is slower. This figure explores how the rate at which immune escape mutants emerge in HLA matched hosts affects the impact of a five-epitope vaccine delivered to the all hosts in the population (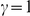) 50 years into an epidemic. Three different escape rates are considered: rapid escape (circles 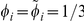 years^−1^ for *i* = 1∶5), slow escape (triangles; 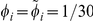 years^−1^ for *i* = 1∶5) and no escape (squares; 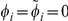 years^−1^ for *i* = 1∶5) at each epitope. In each example, escape occurs at the same rate in vaccinated and unvaccinated hosts (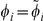 for *i* = 1∶5). Reversion takes an average of 36 years at each epitope in both vaccinated an unvaccinated hosts (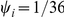 years^−1^ for *i* = 1∶5). A) shows the proportion of hosts with uncontrolled HIV. B) shows the proportion of hosts infected with HIV. C) shows the escape prevalence amongst HLA-matched hosts. The impact of vaccination (red lines) is compared to the scenario where vaccination is absent (black lines). This figure shows that the prevalence of escape amongst HLA-matched hosts at each epitope would be lower (C) and the vaccine would be more effective in reducing the prevalence of uncontrolled HIV (A) and HIV infection (B) if the rate of escape were slower. The assumptions and parameters used in these figures are the same as those described for Figure 2 except that the infectiousness and life expectancy of successfully vaccinated hosts are fixed at 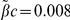 and 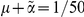, respectively (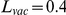). Note that in this figure and the remaining figures, different markers (e.g. circles, triangles and squares) are used to distinguish between different model outputs. These do not represent data.

The rate at which escape mutants are selected will therefore be crucial to the impact of a vaccine. If escape mutants abolish effective CTL responses, the more rapidly escape mutants are selected, the more prevalent they would become in the population (Figure 3C) and the less impact the vaccine would have on the prevalence of uncontrolled infection (Figure 3A) and infection (Figure 3B) in the population. In the absence of escape, or if escape is very slow indeed, the reduction in infectiousness conferred by the vaccine could be sufficient to lead to eradication of HIV (Figure 3B, squares). These dynamics are explored for different escape rates: fast escape (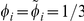 years^−1^ for *i* = 1∶5), slow escape (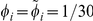 years^−1^ for *i* = 1∶5) and no escape (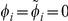 years^−1^ for *i* = 1∶5) at each epitope.

The results presented throughout assume that at each epitope escape occurs at the same rate in vaccinated hosts as in unvaccinated hosts (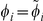 for *i* = 1∶5). However we note that this assumption is largely speculative. It is possible that escape could occur more slowly or more rapidly under vaccination. One hypothesis suggests that because vaccine induced responses will be more effective than natural responses they will impose a greater selective pressure on the virus and drive escape more rapidly. Evidence for this comes from studies in which inferred escape mutants were associated with lower viral loads [49], [50] and the presence of protective HLA class I alleles [51], [52]. Rapid escape has also been observed in some vaccinated macaques that were experimentally infected with a pathogenic hybrid simian-human immunodeficiency virus (SHIV) [19]. An alternative theory suggests that early potent immune responses could curtail viral replication to such low levels that *de novo* generation of escape mutants will be restricted and thus will emerge at a slower rate than observed under natural infection. The reduced emergence of drug resistance with combination antiretroviral therapy compared to single therapy [53] and with high drug adherence compared to low adherence [54], [55] provides support for this hypothesis.

Escape rates under vaccination could therefore plausibly take a range of speeds from very rapid to not at all and could be coupled or uncoupled to the rates observed under natural infection. This does not qualitatively change our finding that vaccine impact will be lower when escape mutants are more prevalent. Vaccine impact is a decreasing function of both the rate of escape in vaccinated hosts (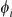) and the rate of escape in unvaccinated hosts (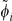). However, it could dramatically change our quantitative findings. This is demonstrated in Figure S2 in which we assume that escape rates in vaccinated hosts are proportional to those in unvaccinated hosts (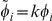 for *i* = 1∶5) but vary the scaling factor (*k = 1/100, 1/10, 1, 10*). If escape were markedly slower in vaccinated compared to unvaccinated hosts, escape prevalence could be much lower and vaccine impact could be much greater. If escape were faster, vaccine impact could be reduced compared to our original estimates.

Escape rates could also be influenced by the breadth of the functional immune response. For example, if escape rates are linked to viral replication rates, a broader CTL response could lead to slower escape rates. This is akin to escape occurring more slowly in hosts receiving combination antiretroviral therapy compared to single therapy [53]. Whilst we have not modelled this possibility, it is clear that it would also affect our quantitative results. As vaccine breadth is increased, the population prevalence of escape would be reduced and the impact of the vaccine would be increased.

**Figure 4:**
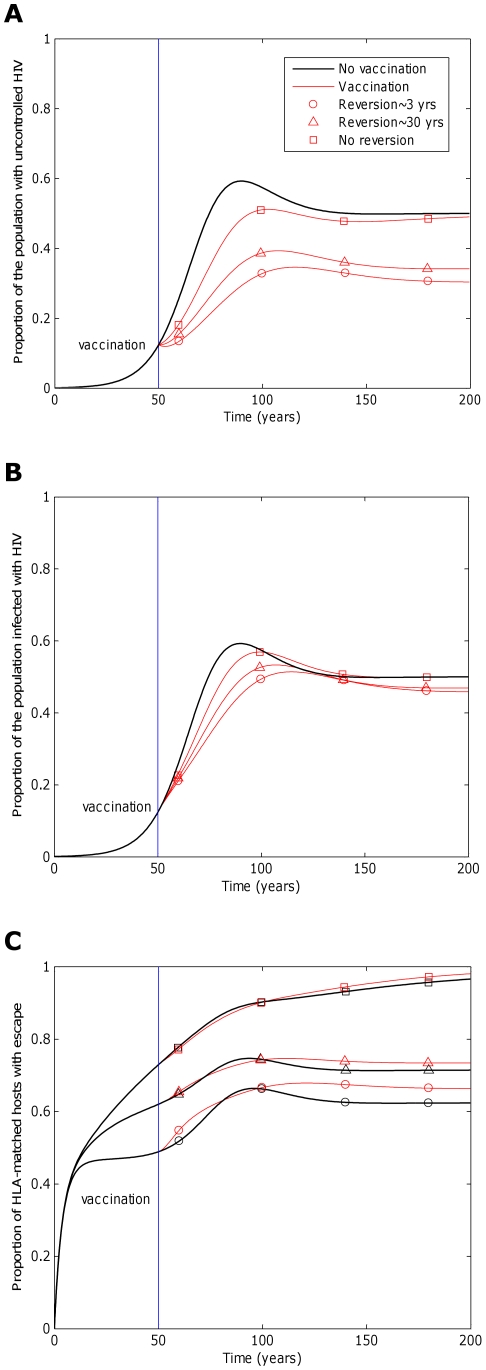
The impact of a vaccine will be greater if the rate at which immune escape mutants revert in HLA mismatched hosts is faster. This figure explores how the rate at which immune escape mutants revert in HLA mismatched hosts affects the impact of a five-epitope vaccine delivered to the population 50 years into an epidemic. Model simulations are presented for different reversion rates: rapid reversion (circles; 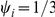 years^−1^ for *i* = 1∶5), slow reversion (triangles; 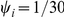 years^−1^ for *i* = 1∶5) and no reversion (squares; 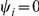 years^−1^ for *i* = 1∶5) at each epitope in both vaccinated and unvaccinated hosts. Escape takes an average of 8 years at each epitope in both vaccinated an unvaccinated hosts (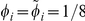 years^−1^ for *i* = 1∶5). A) shows the proportion of hosts with uncontrolled HIV. B) shows the proportion of hosts infected with HIV. C) shows the escape prevalence amongst HLA-matched hosts. The impact of vaccination (red lines) is compared to the scenario where vaccination is absent (black lines). This figure shows that the prevalence of escape amongst HLA-matched hosts at each epitope would be lower (C) and the vaccine would be more effective in reducing the prevalence of uncontrolled HIV (A) and HIV infection (B) if immune escape mutants revert more rapidly in HLA mismatched hosts. The assumptions and parameters used in these figures are the same as those described for Figure 2 except that the infectiousness and life expectancy of successfully vaccinated hosts are fixed at 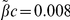 and 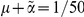, respectively (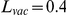).

**Figure 5:**
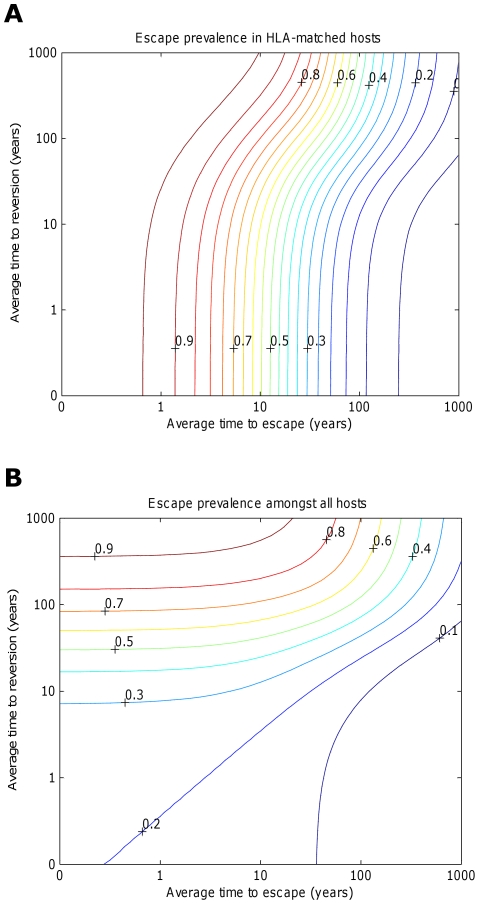
Contour plots showing how the prevalence of escape amongst A) HLA matched hosts and B) all hosts varies according to the escape and reversion rate. These contour plots explore how the prevalence of escape 200 years into an epidemic varies according to the rate of escape in HLA matched hosts and the rate of reversion in HLA mismatched hosts. Vaccination is applied to all hosts in the population 50 years into an epidemic. Escape is assumed to occur at the same rate at each epitope. Reversion is assumed to occur at the same rate at each epitope and revert at the same rate. At each epitope, escape rates in vaccinated and unvaccinated HLA-matched hosts are assumed to be equal (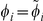 for *i* = 1∶5). The escape and reversion rates are presented in terms their reciprocal – the average time to escape (*x*-axis) and average time to reversion (*y*-axis). The contours show that the prevalence of escape amongst A) HLA-matched hosts and B) all hosts increases as the rate of escape increases and the rate of reversion decreases; however, the increase in escape prevalence in HLA-matched hosts with reversion rate is limited when revision takes an average of 10 years or less. The assumptions and parameters used in these figures are the same as those described for Figure 2 except that the infectiousness and life expectancy of successfully vaccinated hosts are fixed at 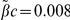 and 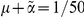, respectively (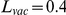).

The fitness costs that escape mutants impose on the virus will also affect the impact of a vaccine. Fitness costs can lead to reversion of escape mutants following transmission to HLA mismatched hosts. If escape mutants are stable they would continuously accumulate in the population until fixation (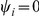 years^−1^ for *i* = 1∶5; Figure 4C, squares), eventually rendering a vaccine useless for future generations (Figure 4A and 4B, squares). However, this could take several decades. If instead, escape mutants revert in HLA-mismatched hosts they would not increase indefinitely, but would reach a prevalence that is determined by the rate of selection and reversion of escape mutants (Figure 4C and Figure 5). The escape prevalence would be lower if reversion is faster. A vaccine would therefore have more impact in reducing the prevalence of uncontrolled infection (Figure 4A) and infection (Figure 4B) in the population if escape mutants confer a greater fitness cost to the virus and revert more rapidly in HLA mismatched hosts.

Taken alone the inferences from our analysis suggest that regions of the HIV genome that remain more conserved (i.e. escape slowly and revert quickly) under natural immune responses would make the best targets for a vaccine. However, it is important to highlight that moderating the effects of CTL escape is not the only consideration for a vaccine. The targeted genomic region must also be able to elicit potent immune responses in the first instance. It is not a given that regions that are most robust to escape are also the most potent. For example pol is the most conserved of the HIV genes and yet pol-specific responses did not emerge as statistically associated with lowering viremia in a recent study [32]. In that study, only gag-specific responses displayed such an association. A second investigation [51] has also revealed that epitopes with the highest prevalence of inferred escape mutants are mostly restricted by HLA alleles that confer a survival advantage. This suggests that the best regions of the genome for eliciting responses under vaccination may actually be those that elicit natural responses that are strong enough to drive CTL escape and hence are less conserved than other regions. The best regions for a vaccine would therefore be the ones that balance the need to elicit effective responses with the detrimental effect of CTL escape.

### Should a vaccine span a broad or narrow range of CTL epitopes?

**Figure 6:**
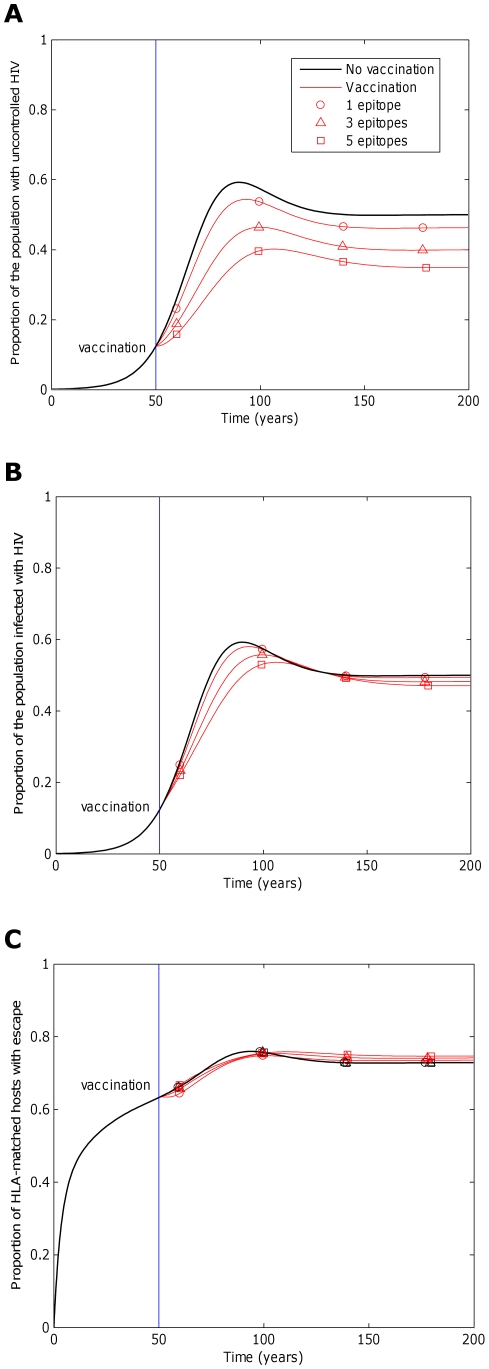
The impact of a vaccine would increase with vaccine breadth. This figure explores how breadth of a vaccine would affect its impact when delivered to a population 50 years into an epidemic. Model simulations are presented for vaccines that elicit responses to one epitope (circles), three epitopes (triangles) and 5 epitopes (squares). Escape takes an average of 8 years at each epitope in both vaccinated an unvaccinated hosts (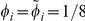 years^−1^ for *i* = 1∶5). Reversion takes an average of 36 years at each epitope in both vaccinated an unvaccinated hosts (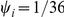 years^−1^ for *i* = 1∶5). A) shows the proportion of hosts with uncontrolled HIV. B) shows the proportion of hosts infected with HIV. C) shows the escape prevalence amongst HLA-matched hosts. The impact of vaccination (red lines) is compared to the scenario where vaccination is absent (black lines). C) shows that the prevalence of escape at each epitope in HLA-matched hosts would be relatively invariant to the number of epitopes included in the vaccine. Two conflicting forces that result from the increase in protection offered by additional epitopes affect this prevalence. More protection means that hosts live longer so have more time to select escape mutants, but also means that hosts are less infectious, so are less likely to transmit mutants to other hosts. Despite the lack of variation of escape prevalence (at each epitope) with vaccine breadth, vaccines that elicit broader responses would be markedly more effective at reducing disease (A) because they would ensure that more hosts have the potential to recognise at least one epitope and more hosts remain protected by alternative responses if escape occurs in one epitope. Infection prevalence would also reduce with increased vaccine breadth (B), but to a lesser extent. The assumptions and parameters used in these figures are the same as those described for Figure 2, except that the infectiousness and life expectancy of successfully vaccinated hosts are fixed at 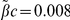 and 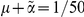, respectively (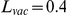).

We have investigated how the impact of a vaccine could be affected by the number of epitopes to which the vaccine elicits responses. Using this model we predict that a vaccine would be more effective if it elicits responses to a larger number of epitopes. Because each epitope is recognised by only a fraction of all hosts, the more epitopes included in the vaccine, the larger the average number of vaccine-elicited responses per host. It is plausible that protection offered by multiple epitopes is additive, so hosts who recognise several epitopes could control virus better than hosts who recognise a single epitope. Our model, however, assumes that a host's viral control is the same regardless of whether a single epitope or multiple epitopes are recognised. Even under this more conservative ‘all or nothing’ assumption, a vaccine that elicits more responses would be more effective in reducing uncontrolled infections (Figure 6A). More epitopes means that more hosts recognise at least one epitopes, so have some protection, and that more hosts remain protected by alternative responses if escape occurs in one of the epitopes they restrict. The benefits associated with a broader response are seen despite relative invariance with vaccine breadth in the escape prevalence amongst HLA-matched hosts at each epitope (Figure 6C). Two conflicting forces that result from the increase in protection offered by additional epitopes affect the prevalence of escape. Broader protection means that hosts live longer so have more time to select escape mutants in any particular epitope. However, this is outweighed by the fact that broader protection also means that infectiousness is reduced and escape mutants are transmitted onto other hosts less frequently. Infection prevalence reduces only to a small extent with increased vaccine breadth (Figure 6B).

It is important to highlight that these results assume that epitopes act independently. One component of this assumption is that escape rates at each epitope are independent of the presense of escape mutants at other epitopes and are therefore independent of the contribution that other epitopes make to viral control. As mentioned previously, if escape rates were linked to viral replication rates, a broader vaccine could lead to slower escape rates. This could markedly reduce the escape prevalence at each epitope and improve the impact of the vaccine compared to the simulations presented here.

The results presented also assume that epitope-specific immune responses are not in competition with each other. Each response is either beneficial or neutral to viral control. Our assumption that epitopes do not compete is based upon results from a large study of competition between CTL responses [56]. However, if epitopes do compete and some responses are more effective than others, then increasing the breadth of a vaccine could potentially be detrimental to the impact of a vaccine.

### Should a vaccine target epitopes restricted by rare or common HLA alleles?

**Figure 7:**
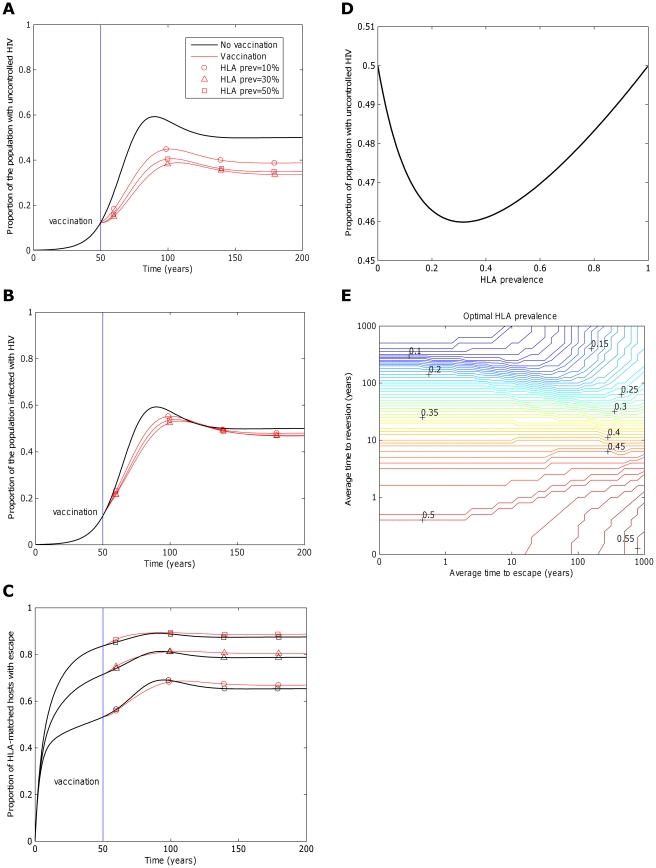
The impact of a vaccine varies with the fraction of the population that are HLA-matched for each epitope. Panels A), B) and C) explore how the frequency with which epitopes are recognised in the population affects the impact of a five-epitope vaccine delivered to the population 50 years into an epidemic. Model simulations are presented under different assumptions about the fraction of the population that is HLA match for each of the five epitopes: 10% (circles), 30% (triangles) and 50% (squares). Escape takes an average of 8 years at each epitope in both vaccinated an unvaccinated hosts (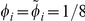 years^−1^ for *i* = 1∶5). Reversion takes an average of 36 years at each epitope in both vaccinated an unvaccinated hosts (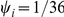 years^−1^ for *i* = 1∶5). A) shows the proportion of hosts with uncontrolled HIV. B) shows the proportion of hosts infected with HIV. C) shows the escape prevalence amongst HLA-matched hosts. The impact of vaccination (red lines) is compared to the scenario where vaccination is absent (black lines). C) reveals that the prevalence of escape amongst HLA-matched hosts at each epitope would be higher if the restricting HLA is more prevalent. However, escape prevalence is not the only factor which contributes to vaccine impact since epitopes restricted by more prevalent HLAs will also provide protection to a greater fraction of the population. A) and B) reveal that an intermediate HLA prevalence (30% in this example, triangles) can optimise vaccine impact. This result is demonstrated more explicitly in panel D) in which the prevalence of uncontrolled infection for the single-epitope version of the model 200 years into an epidemic is used to explore the effect of HLA prevalence upon vaccine impact . D) shows that uncontrolled infection prevalence is a u-shaped function of the restricting HLA prevalence, thus is minimised at intermediate HLA prevalences. In this example (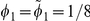 year^−1^ and 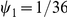 years^−1^) vaccine impact is optimised when 32% of the population are HLA matched for the epitope. E) shows that the HLA prevalence that maximises vaccine impact 200 years into an epidemic is dependent upon the rate of escape in HLA matched hosts (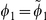) and rates of reversion in HLA mismatched hosts (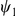). The assumptions and parameters used in these figure are the same as those described for Figure 2, except that the infectiousness and life expectancy of successfully vaccinated hosts are fixed at 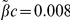 and 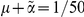, respectively (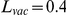).

Whilst we have demonstrated that in theory a vaccine should elicit as broad a response as possible, engineering limitations could restrict breadth in practise. In such case, it is pertinent to ask whether it is preferable to target epitopes that will be recognised by a small or large fraction of the population. In other words, should a vaccine target epitopes restricted by rare or common HLA types. This question is non-trivial because an epitope restricted by a common HLA will span a larger fraction of the population, but will also accrue escape mutants more rapidly. We explore this question in Figure 7, where the dynamics of three vaccines, targeting differentially prevalent HLAs, are explored. One example vaccine elicits responses to five epitopes, each recognised by 10% of the population (circles). In the remaining two examples, each epitope is recognised by 30% (triangles) and 50% (squares) of the population, respectively. Figure 7C indicates that, all else being equal, the prevalence of escape at each epitope amongst HLA matched hosts increases as the HLA prevalence increases. This ensures that the impact of the vaccine does not continuously increase with HLA prevalence. As HLA prevalence initially increases, impact improves because of greater coverage (Figures 7A and 7B; the 30% vaccine (triangles) is more effective than the 10% vaccine (circles)); however, as prevalence increases further, the detrimental effects of escape eventually outweigh the increase in coverage (Figures 7A and 7B; the 50% vaccine (squares) is less effective than the 30% vaccine (triangles)). This result is demonstrated more explicitly in Figure 7D in which the proportion of hosts with uncontrolled infection 200 years into an epidemic after vaccination (50 years into an epidemic) with a single epitope is shown to be a u-shaped function of the prevalence of the restricting HLA at that epitope. Thus, an intermediate HLA prevalence optimises vaccine impact. For example, for an epitope that escapes at a rate of 1/8 years^−1^ and reverts at rate of 36 years^−1^, a restricting HLA prevalence of 32% will optimise vaccine impact (Figure 7D).

Optimal HLA prevalence is not the same for all epitopes, though. It is higher if reversion is slower and it varies with the rate of escape (Figure 7E). Furthermore, it can also be affected by the nature of the other epitopes that are present in the vaccine (i.e. their escape rates, reversion rates and restricting HLA prevalences). In the context of our simple model – which assumes that epitopes act independently – the effect of other epitopes upon the optimal HLA prevalence at any particular epitope is small (Figure S3). However, it is clear that if the impact of different epitopes upon viral control is heavily interdependent, such as in the presence of competition between CTLs, the optimal HLA prevalence for any particular epitope could be greatly influenced by the nature of the other epitopes in the vaccine. As an example, consider an epitope which elicits a CTL response that contributes nothing or very little to viral control and yet competes strongly with effective CTLs directed against other epitopes. The optimal HLA prevalence would be very low, irrespective of the rate at which escape and reversion occur in that epitope.

In summary, epitopes that optimise vaccine impact tend to escape slowly, revert quickly and are restricted by intermediately prevalent HLAs. However it is the combination of each of these factors and the nature of interdependence between epitopes that determines which epitopes will be optimal.

### The control reproductive number

To investigate the impact of a vaccine on an HIV epidemic theoretically and to confirm our numeric interpretation of the model, we have determined the control reproductive number (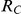) for the one-epitope (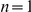) version of this system (Text S2 and Figure 1). The term 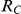 is analogous to the basic reproductive number (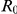) – the expected number of secondary cases caused by one primary case in a *wholly* susceptible population – but it accounts for a portion of the population being resistant to infection because of a control strategy, in this case vaccination. Thus 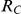 can be regarded as the expected number of secondary cases caused by one primary case in a wholly uninfected population under a control strategy. It is perhaps worth highlighting here, that whilst 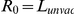, 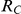 is not equal to 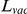. Whereas 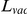 describes the lifetime transmission potential of a specific *host type* (vaccinated hosts), 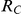 describes the average lifetime transmission potential of an average individual in a *population* – some of whom are vaccinated and some of whom are not. In the simulations presented here we have assumed that vaccination does not confer any level of sterilizing immunity (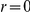). However, for completeness, for the analysis of the control reproductive number, we use the more general version of our model in which a fraction (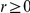) of vaccinated hosts wholly are resistant to infection.

In a system that has only one infection state, the expected number of secondary cases from one primary case has a simple meaning since there is only one type of primary case and one type of secondary case. In the single-epitope version of our model, however, there are eight different infection states and the expected number of secondary cases from one primary case is dependent upon the state of the primary case. Thus 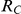 can be defined more precisely to be the expected number of secondary cases produced by a *typical* individual in a wholly uninfected population, where by ‘typical’ we mean an individual whose state is distributed according to the average distribution of hosts amongst the different states. Another way of phrasing this is that is that 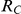 is the average per-generation multiplication number [57].

Under this definition, 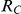 can be found by calculating the largest eigenvalue of the next generation matrix, **K**, where the elements of **K**, 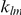, are the average number of individuals of infectious state *l*, created when a single individual of state *m* is introduced into the population [57]. In our model there are eight infectious states, however the model can be collapsed into a minimum of five states. 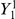 and 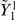 can be combined into a single state, 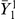 (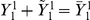), which represents infected HLA-matched hosts with escape, and similarly for the pairs 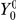 and 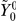, and 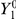 and 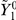. The next generation matrix of the five states 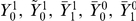 is provided in Text S3.

The analytic formula for the largest eigenvalue of this matrix, although it can be written out explicitly, is too complex to yield insight so is not presented. However, the control reproductive number (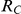) for this system can be calculated numerically for specific parameter values. Some example values are given in Text S4. These examples confirm the dynamic model outputs. They show that a CTL-based vaccine would have more impact upon an epidemic – i.e. 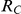 would be lower – if escape in vaccinated (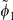) and unvaccinated (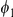) HLA matched hosts is slower, reversion in HLA mismatched hosts (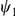) is faster, the transmission coefficient of successfully vaccinated hosts (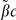) is lower, the life expectancy of successfully vaccinated hosts (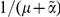) is shorter, the proportion of hosts who are vaccinated (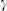) is higher and the fraction of vaccinated hosts resistant to infection is greater (*r*). Furthermore, 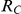 a u-shaped function of HLA prevalence (*p* ^1^) and 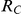 is lower if for unvaccinated (or unsuccessfully vaccinated) hosts the transmission coefficient (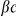) is smaller or the life expectancy (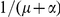) is shorter. Finally, assuming that the life expectancy of infected hosts is fixed (i.e. 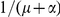 and 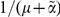 are both fixed), 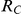 is invariant to the life expectancy of uninfected hosts (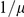).

## Discussion

We have explored the impact of a CTL-inducing vaccine that reduces infectiousness and extends life expectancy upon the prevalence of infection and uncontrolled infection in the population. Specifically, we have explored how the impact of such a vaccine would be affected by the spread of escape mutants, the breadth of the induced CTL response and the fraction of the population that recognises each epitope in the vaccine.

Firstly, our model has shown that vaccination could reduce the prevalence of uncontrolled infection and could also reduce the prevalence of infection by reducing the infectiousness of infected hosts. Precisely whether a vaccine would reduce infection prevalence, however, is dependent not only upon the extent to which it reduces infectiousness, but also the extent to which it extends life expectancy and thus extends the period over which transmission can occur. A CTL inducing vaccine could actually increase infection prevalence if the reduction in infectiousness is more than outweighed by the increase in longevity, but any increase is likely to be marginal. Furthermore data from natural infection concerning the relationship between infectiousness, life expectancy and viral set point suggests that vaccination is most likely to reduce overall transmission potential by a factor of at least five and therefore reduce infection prevalence.

Secondly, our model has shown that the impact of a CTL-inducing vaccine could be significantly reduced by CTL escape, particularly if escape is rapid and mutants confer low fitness costs, so revert only slowly in HLA mismatched hosts. The model therefore suggests that a vaccine would be more robust to escape if it spans a conserved, functional region of the genome. It is important to acknowledge, however, first and foremost, that a vaccine must be able to elicit effective immune responses. The most conserved regions of the genome are not necessarily able to induce the most effective responses. For example pol is the most conserved of the HIV genes and yet in recent study pol-specific responses were not associated with lowering viremia [32]. The best vaccine would therefore be one which weighs up the need to elicit effective responses with the detrimental effect of CTL escape.

Thirdly, our model has shown that in the absence of competition between CTL responses, a vaccine would be more effective if it spans a broader range of CTL epitopes. Such a vaccine would provide protection for a larger proportion of hosts and would also be more robust to the effects of CTL escape.

Finally, we have shown that the frequency with which each epitope is recognised in the population will affect vaccine impact. An epitope restricted by a prevalent HLA will span a greater fraction of the population, but will also accumulate escape mutants more rapidly. Thus epitopes restricted by an intermediate fraction of the population tend to optimise vaccine impact. However, the precise restricting HLA prevalence for any epitope that optimises vaccine impact is dependent upon the rate at which mutants escape and revert at that epitope, as well as the nature of other epitopes included in the vaccine. In a scenario where vaccine breadth is limited, the choice of *which* epitopes to include in the vaccine should therefore account for the combined effects of escape rates, reversion rates and restricting HLA prevalences of different epitopes.

We have deliberately kept our model simple to allow a transparent explanation of what assumptions we have made. However, it is not difficult to propose ways in which the emergence of escape mutants in a vaccinated population is more complicated than is represented by our model. One of the main assumptions made in our simulations is that the vaccine does not provide any level of sterilizing immunity (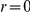). Following the partial success of the Thai vaccine trial in which HIV incidence was reduced by 31% over the first three years following inoculation [15], it has become less likely that an HIV vaccine with no ability to prevent infection will be made widely available. Although the primary concern of our study was a non sterilizing CTL based vaccine, our model is also designed to allow for a fraction, *r*, of vaccinated hosts to be completely resistant to infection. In the results discussed throughout this paper we have assumed that 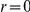. However, as demonstrated by our analysis of the control reproductive number (Text S4), infection prevalence would decreases as the fraction of vaccinated hosts resistant to infection (*r*) increases. Indeed, irrespective of CTL escape and reversion rates, a vaccine with a neutralising component could lead to eradication of HIV if the fraction of hosts resistant is sufficiently large.

Our model also makes many simplifying assumptions about the way in which escape mutants evolve within individuals and transmit between individuals. For example, we assume that escape mutants cannot revert in HLA matched hosts; that escape and reversion rates are homogeneous across the duration of infection; that people are equally infectious throughout their infection and that epitopes are independent entities which do not overlap each other. We have previously explored the impact of these assumptions on the spread of escape mutants in an unvaccinated population [22]. We have shown that such factors could change the pace of the spread of escape mutants in the population, but would not qualitatively change our findings. Likewise, in the context of a vaccinated population, as studied here, our main findings are robust to these assumptions. We have also assumed that individuals are infectious with only one viral strain at a time. In reality, individuals harbour a quasispecies of viral strains, yet transmit single or very few virions during each transmission event [58], [59], thus it is not certain that the dominant strain within the transmitter will be passed to the recipient. It is possible that escape mutants could, on average, be transmitted less frequently than predicted by the single strain model. However, this too does not affect our main findings that vaccine impact will be greater if mutants escape slowly, revert quickly and the breadth of the response is greater. Numerically, a reduction in the transmission probability of escape mutants will have a similar effect to an increase in reversion rate.

Other simplifications pertain to the description of the vaccine. For example, we have run our model simulations using a vaccine that contains up to five CTL epitopes. Ideally a vaccine would make responses to more epitopes than just five. In addition, the fraction of the population that respond to most epitopes in a vaccine is likely to be less than the 20% we have assumed in our simulations. The spread of escape at any particular epitope is therefore likely to be a little more sedate than we predict. Furthermore, the number of epitopes that an average individual will make a response to is likely to be much more than the median of just one that our simulations assume. This, combined with a reduction in the spread of escape mutants, could lead to a more effective and robust vaccine than we predict. Indeed, the extent to which a vaccine remains effective in the face of escape is another assumption that we have made. We have assumed that the vaccine will confer protection in all individuals who restrict at least one epitope they are vaccinated against and in which escape mutations are absent. In reality, the level of protection from a vaccine could change in a more progressive manner, i.e. could increase steadily with the number of epitopes providing protection. We would expect that such processes would change our results quantitatively, but not qualitatively.

One assumption that affects our qualitative as well as our quantitative results is that epitopes act independently. Thus, we assume that different epitope-specific immune responses are not in competition with each other. Each response is either beneficial or neutral to viral control. This assumption has important implications because if epitopes do compete and some responses are more effective than others, then increasing the breadth of a vaccine could potentially be detrimental to the impact of a vaccine. Our assumption is based upon results from a large study of competition between CTL responses [56]. Contrary to smaller studies that focussed on a limited number of individuals and/or a limited number of epitopes [60]–[61], this study found no evidence for competition between CTL responses directed against different HIV-1 epitopes. However, we note that there is scope to investigate evidence for competition further with different patient cohorts and more detailed CTL response measurements. In particular, it would be interesting to investigate competition data from post-infection vaccine trials, as this would closely match the experimental manipulation of CTLs that is used to test for competition in non human subjects. If evidence did emerge for competition between CTL responses directed against different epitopes it would change our finding that increasing the breadth of a vaccine is always beneficial. Instead, a vaccine with a relatively limited breadth, focussed on particularly effective responses could be optimal. Likewise, competition would change the dependency of vaccine impact upon the escape rate, reversion rate and restricting HLA prevalence for each epitope.

Our assumption that epitopes act independently also means that the diversity of the functional CTL response has no impact upon escape and reversion rates. The only factor we assume influences escape rates is whether or not the individual is vaccinated. Reversion rates are fixed across both vaccinated and unvaccinated host. It is plausible that a more diverse CTL response could repress viral replication to lower levels, thereby restricting the *de novo* generation of escape mutants and thus reducing escape rates compared to those observed natural infection. A sufficiently broad vaccine could prevent escape all together. An alternative hypothesis suggests that vaccination could increase escape rates compared to natural infection because of a greater selective pressure imposed by more potent responses. Precisely how quickly escape will occur under vaccination therefore remains an open question.

## Methods

The model that we have developed can be described as follows: suppose that the vaccine induces responses to *n* different epitopes. The vector 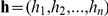 represents the host type, indicating which of the *n* epitopes the host restricts: 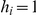 if the host restricts epitope *i* and 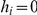 if it does not. Hosts are born into the population at a constant rate 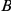, thus birth rates are independent of current host densities. In our simulations we set this parameter to equal to the multiple of the population death rate (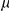) and the total population size at the start of the simulation. Thus, the population size would remain constant in the absence of infection. A fraction, 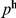, of these newborns are of host type **h**, ensuring that in the absence of vaccination, the fraction of the population with each host type remains fixed. A fraction, 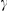, of newborns receive the vaccine at birth and a fraction, *r*, of vaccinated hosts are resistant to infection. At any given time, *t*, each infected host is assumed to be infected with a single viral strain. This strain may or may not carry a mutation at each of the *n* epitopes. The vector 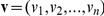 is the virus type, indicating the epitopes at which the virus carries escape mutants: 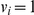 if the virus is mutant at epitope *i* and 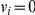 if it is wildtype. 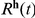, 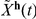 and 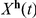 represent the number of vaccinated resistant, vaccinated susceptible and unvaccinated susceptible hosts of type 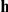 at time *t*, respectively. These hosts each have an average life expectancy of 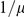. 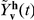 and 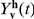 represent the number of vaccinated and unvaccinated type 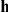 hosts infected with virus type 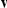 at time *t*, respectively. 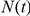 is the total number of hosts in the population at time *t*. Thus 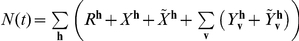. If 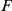 is the set of pairs 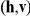 which have 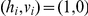 for at least one epitope *i*, then a vaccinated host of type 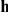 who has virus type **v**, such that 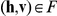, is successfully vaccinated, i.e. they restrict at least one epitope they are vaccinated against and that epitope has no escape mutations. By contrast, a vaccinated host with 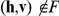 is unsuccessfully vaccinated and has the same properties as an unvaccinated infected host. Text S1 demonstrates the use of this notation in an example that considers a vaccine that contains five epitopes. Successfully vaccinated hosts are less infectious than unvaccinated or unsuccessfully vaccinated infected hosts (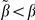), but their average life expectancy is longer (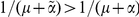; equivalently 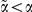). This imposes a limited degree of selection on host HLA genotypes over the course of an infection (e.g. in our simulations an initial HLA prevalence of 20% increases to 21% after 200 years) and has no significant impact upon the dynamics that we explore. We have previously investigated the impact of the HIV epidemic upon HLA gene frequencies amongst the population in absence of vaccination [62]. A variation of our vaccine model in which the birth rate of each host type is dependent upon the current density of that host type in the population could be used to fully explore host-pathogen co-evolution under vaccination, but for brevity we do not explore these dynamics here.

The average rate of partner exchange (*c*) is assumed to be the same for all host types. In line with convention for a sexually transmitting disease, this parameter is included explicitly, despite being mathematically redundant, appearing as a scaling factor of 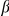 and 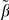 throughout. Overall the force of infection from infected hosts with virus type **v** at time *t* is defined as 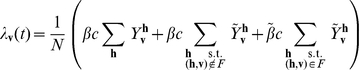, that is the weighted sum of infectiousness from the three host types infected with virus type **v**: unvaccinated, unsuccessfully vaccinated and successfully vaccinated. For hosts who recognise epitope *i* and who are infected with a virus that is wildtype at epitope *i*, the rate of escape at that epitope is 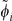 if they are vaccinated and 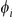 if they are not. Regardless of vaccination, the rate of reversion at epitope *i* is 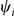 in hosts who don't recognise epitope *i* and are infected with a mutant at epitope *i*. To enable us to write the model equations in a simple and general form we have defined 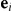 to be the unit vector of length *n* that has *i*th coordinate equal to 1, e.g. 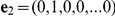; 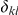 to be the Kronecker delta and 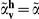 if 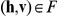 and 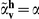 otherwise (Table 3). Using these, the model can be described by the set of coupled ordinary differential equations given in expression (1). Tables 1 and 2 summarise the variables and parameters used in this model.
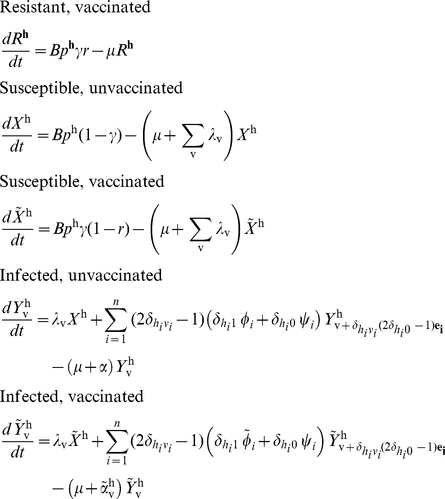
(1)The simplest version of this model, when the vaccine contains only one epitope (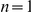), is represented graphically in Figure 1.The model equations for the single-epitope version of the model are presented in Text S2.

## Supporting Information

Figure S1 The impact of vaccination is compared for different epidemic dynamics.
(PDF)

Figure S2 The impact of a vaccine is dependent upon the extent to which vaccination affects the rate at which escape mutants emerge.
(PDF)

Figure S3 The optimal HLA prevalence at any particular epitope can be affected by the nature of the other epitopes included in the vaccine.
(PDF)

Text S1 An illustration of how it is determined whether a host is successfully vaccinated or not. This demonstrates the use of the notation *F* to describe the set of host and virus types that are successfully vaccinated.
(PDF)

Text S2 Ordinary differential equations describing the single epitope version (n = 1) of the model.
(PDF)

Text S3 The next generation matrix for the single epitope version of the model.
(PDF)

Text S4 The impact that different parameters have upon the control reproductive number, *R_C_*, for the single epitope version of the vaccine (*n* = 1).
(PDF)
