# Supplementary material for: Modelling the Spread of HIV Immune Escape Mutants in a Vaccinated Population
Source: PLoS Comput Biol. 2011 Dec 1;7(12):e1002289. doi: 10.1371/journal.pcbi.1002289 (PMC3228780; doi:10.1371/journal.pcbi.1002289)
Supplement: Figure S1 — The impact of vaccination is compared for different epidemic dynamics. (PDF) [file pcbi.1002289.s001.pdf]

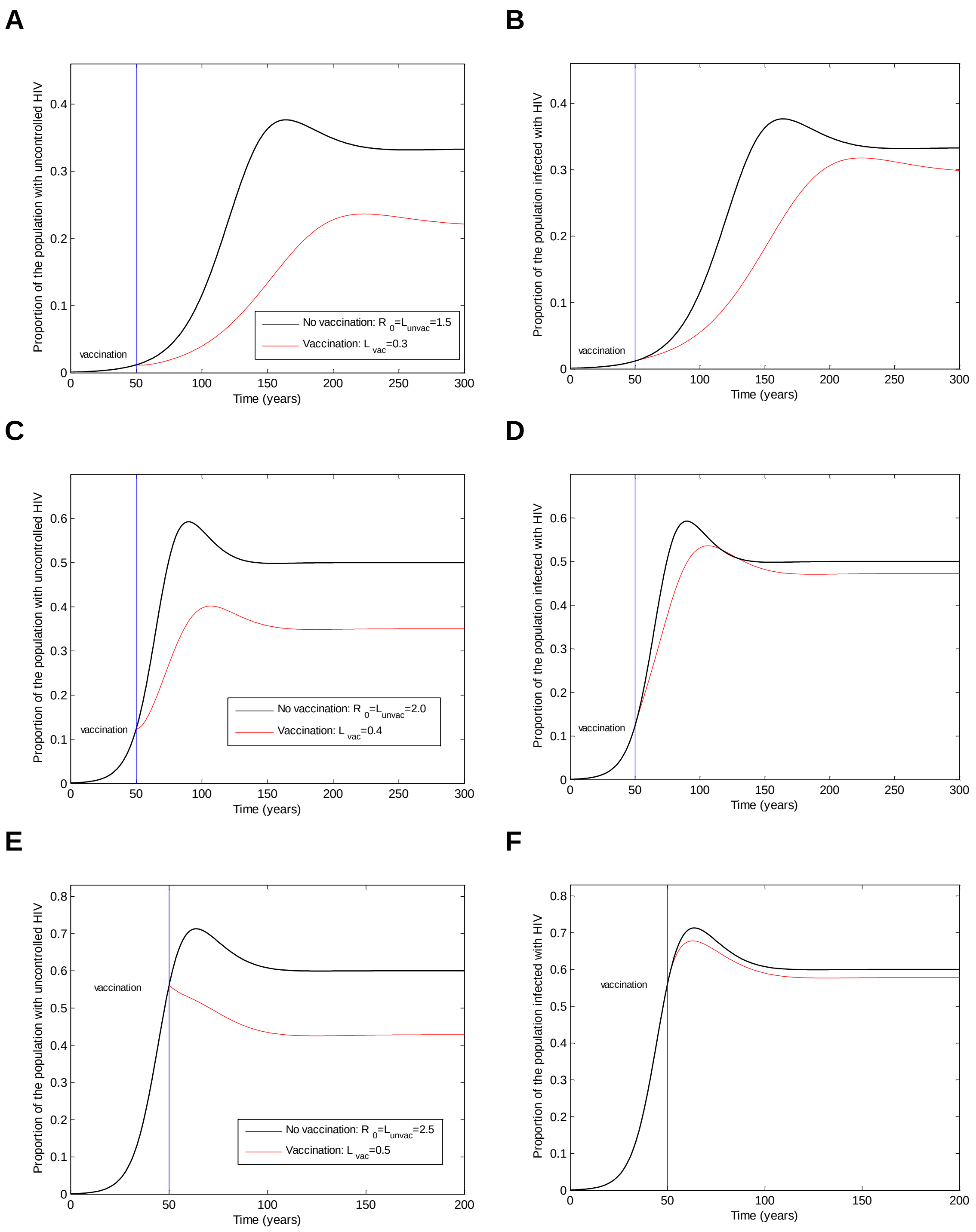


**Figure S1. The impact of vaccination is compared for different epidemic dynamics.**

This figure explores how the impact of vaccination varies according to the underlying epidemic dynamics. The vaccine is delivered to all hosts in the population 50 years into an epidemic. Model simulations are presented for epidemics defined by different basic reproductive numbers: A-B) $R_0 = L_{unvac} = 1.5$ ($\beta c = 0.15$); C-D) $R_0 = L_{unvac} = 2.0$ ($\beta c = 0.20$); and E-F) $R_0 = L_{unvac} = 2.5$ ($\beta c = 0.25$). In each example the life expectancy of infected hosts is fixed at 10 years in vaccinated hosts

($\mu+\alpha=1/10$ years$^{-1}$) and 50 years in unvaccinated hosts ($\mu+\alpha=1/50$ years$^{-1}$). In each simulation vaccination is assumed to cause a fivefold reduction in lifetime transmission potential ($L_{unvac}/L_{vac}=5$; $\beta/\tilde{\beta}=25$). A), C) and E) show the proportion of hosts with uncontrolled infections. B), D) and F) show the proportion of hosts infected with HIV. The impact of vaccination (red lines) is compared to the scenario where vaccination is absent (black lines). This figure shows that in the long-term the *relative* impact of vaccination remains largely invariant to the underlying epidemic dynamics. However, in the short-term, the relative impact does vary with the underlying epidemic dynamics. To highlight this observation we have plotted the results for the three different $R_0$ values over different ranges. Note that in these figures we have assumed 0.1% of the population are infected at the start of the epidemic. Assuming a different initial infection prevalence would change the time it takes for the epidemic to peak, but it would not affect the long term results. The assumptions and parameters used in these figure are the same as those described for Figure 2.
