# Supplementary material for: Modelling the Spread of HIV Immune Escape Mutants in a Vaccinated Population
Source: PLoS Comput Biol. 2011 Dec 1;7(12):e1002289. doi: 10.1371/journal.pcbi.1002289 (PMC3228780; doi:10.1371/journal.pcbi.1002289)
Supplement: Figure S2 — The impact of a vaccine is dependent upon the extent to which vaccination affects the rate at which escape mutants emerge. (PDF) [file pcbi.1002289.s002.pdf]

A

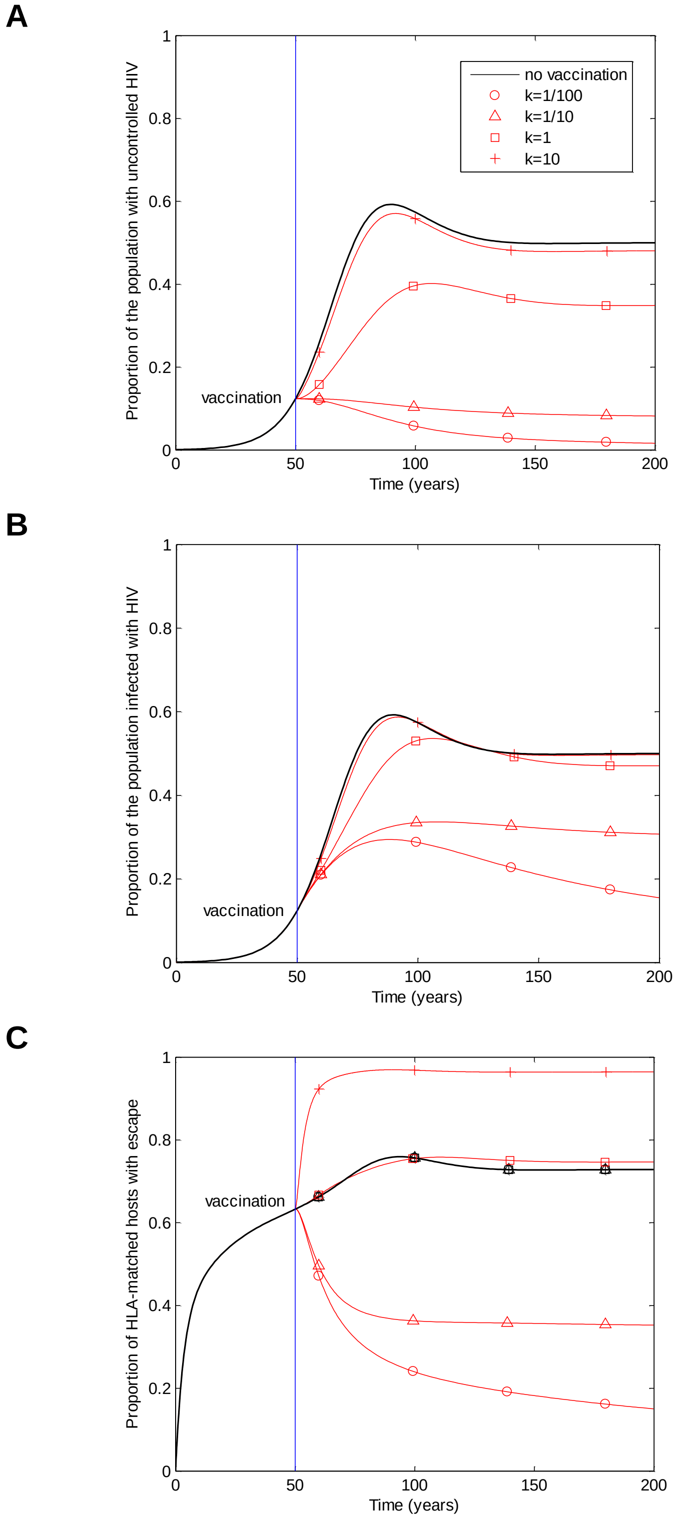

Figure S2. The impact of a vaccine is dependent upon the extent to which vaccination affects the rate at which escape mutants emerge.

This figure explores how the extent to which vaccination affects escape rates affects the impact of a vaccine delivered to a population, 50 years into an epidemic. Throughout this analysis we assume that in the absence of vaccination, the average

time to escape at each epitope is 8 years ($\phi_i = 1/8$ years^{-1} $\forall_i$). However, we vary the rate of escape in the unvaccinated hosts. Specifically, we vary the proportional relationship between the rate of escape in vaccinated and unvaccinated hosts (i.e. we vary k where $\tilde{\phi}_i = k\phi_i$) in terms of 10-fold changes ($k = 1/100, 1/10, 1, 10$). A) shows the proportion of hosts with uncontrolled HIV. B) shows the proportion of hosts infected with HIV. C) shows the escape prevalence amongst HLA-matched hosts at each epitope. The impact of vaccination (red lines) is compared to the scenario where vaccination is absent (black lines). This figure shows that if escape is markedly slower in vaccinated compared to unvaccinated hosts ($k = 1/100$ or $1/10$) escape prevalence (C) could be much lower and vaccine impact (A and B) could be much greater than predicted by our original estimates. If escape is faster ($k = 10$), vaccine impact could be reduced compared to our original estimates ($k = 1$). The remaining assumptions and parameters used in these figures are the same as those described for Figure 2 except that the infectiousness and life expectancy of successfully vaccinated hosts are fixed at $\tilde{\beta}c = 0.008$ and $\mu + \tilde{\alpha} = 1/50$, respectively ($L_{vac} = 0.4$).
