# Supplementary material for: Modelling the Spread of HIV Immune Escape Mutants in a Vaccinated Population
Source: PLoS Comput Biol. 2011 Dec 1;7(12):e1002289. doi: 10.1371/journal.pcbi.1002289 (PMC3228780; doi:10.1371/journal.pcbi.1002289)
Supplement: Figure S3 — The optimal HLA prevalence at any particular epitope can be affected by the nature of the other epitopes included in the vaccine. (PDF) [file pcbi.1002289.s003.pdf]

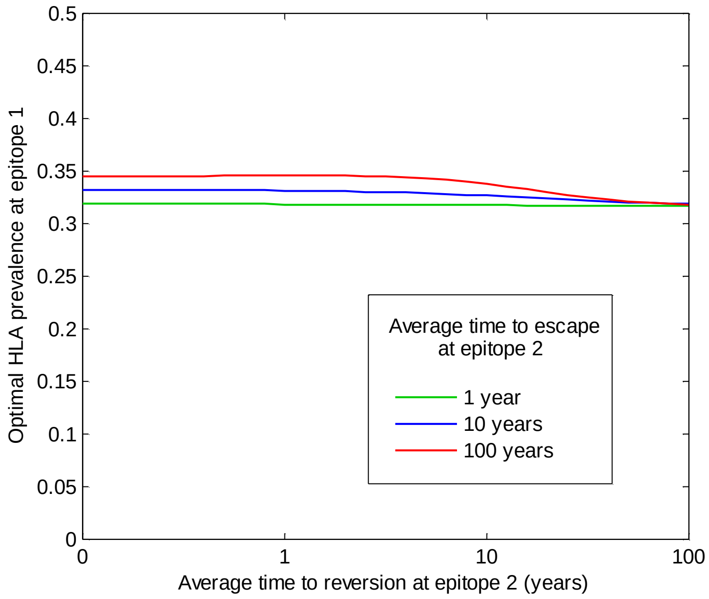

Figure S3. The optimal HLA prevalence at any particular epitope can be affected by the nature of the other epitopes included in the vaccine.

This analysis considers a vaccine that elicits responses to two epitopes. At epitope 1 the average time to escape is fixed at 8 years ($\phi_1 = \tilde{\phi}_1 = 1/8$ years^{-1}) and the average time to reversion is fixed at 36 years ($\psi_1 = 1/36$ years^{-1}). At the second epitope we vary the rate of escape and reversion, but fix the HLA prevalence at 30%. This plot reveals that the optimal HLA prevalence at epitope 1 (*y*-axis) can be affected by the escape and reversion parameters defining epitope 2. This is because the nature of other epitopes in the vaccine affects the fraction of infected hosts who control infection. Those who control infection have a longer life expectancy and transmit less often compared to unvaccinated/unsuccessfully vaccinated hosts. This can affect the prevalence of escape at the epitope 1 and therefore the optimal HLA prevalence at epitope 1. The remaining assumptions and parameters used in these figures are the same as those described for Figure 2 except that the infectiousness and life expectancy of successfully vaccinated hosts are fixed at $\tilde{\beta}c = 0.008$ and $\mu + \tilde{\alpha} = 1/50$, respectively ($L_{vac} = 0.4$).
