# Supplementary material for: Modelling the Spread of HIV Immune Escape Mutants in a Vaccinated Population
Source: PLoS Comput Biol. 2011 Dec 1;7(12):e1002289. doi: 10.1371/journal.pcbi.1002289 (PMC3228780; doi:10.1371/journal.pcbi.1002289)
Supplement: Text S1 — An illustration of how it is determined whether a host is successfully vaccinated or not. This demonstrates the use of the notation F to describe the set of host and virus types that are successfully vaccinated. (PDF) [file pcbi.1002289.s004.pdf]

Text S1. An illustration of how it is determined whether a host is successfully vaccinated or not. This demonstrates the use of the notation *F* to describe the set of host and virus types that are successfully vaccinated.

Consider a vaccine that contains 5 epitopes ($n = 5$) restricted by 5 different HLA types:

Epitope number	1	2	3	4	5
HLA class I restriction	A1	A13	B51	B27	B7

An individual with HLA type A1, A2, B27, B8 would be host type $\mathbf{h_a} = (1,0,0,1,0)$.
An individual with HLA type A1, A13, B51, B27 would be host type $\mathbf{h_b} = (1,1,1,1,0)$.

A virus with no escape mutations in any of the 5 epitopes would be virus type $\mathbf{v_a} = (0,0,0,0,0)$.
A virus with escape in the A1 and B27 epitopes would be virus type $\mathbf{v_b} = (1,0,0,1,0)$.

The set *F* contains ($\mathbf{h_a}, \mathbf{v_a}$), ($\mathbf{h_b}, \mathbf{v_a}$) and ($\mathbf{h_b}, \mathbf{v_b}$) but not ($\mathbf{h_a}, \mathbf{v_b}$).

Host type<br>Virus type	$\mathbf{h_a} = (1, 0, 0, 1, 0)$<br>$\mathbf{v_a} = (0,0,0,0,0)$	$\mathbf{h_a} = (1,0,0,1,0)$<br>$\mathbf{v_b} = (1,0,0,1,0)$	$\mathbf{h_b} = (1, 1, 1, 1, 0)$<br>$\mathbf{v_a} = (0,0,0,0,0)$	$\mathbf{h_b} = (1, 1, 1, 1, 0)$<br>$\mathbf{v_b} = (1,0,0,1,0)$
Ticks and crosses representing the epitopes that do and don't provide protection	(√, ×, ×, √, ×)	(×, ×, ×, ×, ×)	(√, √, √, √, ×)	(×, √, √, ×, ×)
In *F*? Hosts are protected if they have protection from at least one epitope.	Yes	No	Yes	Yes

Notice that we assume no difference in the survivorship or infectiousness of an $\mathbf{h_b}$ host whether infected with a $\mathbf{v_a}$ virus or a $\mathbf{v_b}$ virus. Provided one non-escaped epitope is restricted, the host is successfully vaccinated.
