# Supplementary material for: Modelling the Spread of HIV Immune Escape Mutants in a Vaccinated Population
Source: PLoS Comput Biol. 2011 Dec 1;7(12):e1002289. doi: 10.1371/journal.pcbi.1002289 (PMC3228780; doi:10.1371/journal.pcbi.1002289)
Supplement: Text S2 — Ordinary differential equations describing the single epitope version (n = 1) of the model. (PDF) [file pcbi.1002289.s005.pdf]

Text S2. Ordinary differential equations describing the single epitope version (n=1) of the model.

Equations 1-2 describes how the number of resistant hosts in the population changes over time. Equations 3-6 describe how the number of hosts in each of the four susceptible host categories change over time. Equations 7-14 describe how the number of hosts in each of the eight infected host categories change over time. For these equations the force of infection for the wildtype and escape mutant strains is given by $\lambda_0 = \left(\beta c(Y_0^1 + Y_0^0 + \tilde{Y}_0^0) + \tilde{\beta}\tilde{c}\tilde{Y}_0^1\right)/N$ and $\lambda_1 = \beta c(Y_1^1 + Y_1^0 + \tilde{Y}_1^1 + \tilde{Y}_1^0)/N$, respectively, where N represents the total population size, $N = \sum_{h=0,1}\left(R^h + X^h + \tilde{X}^h + \sum_{v=0,1}\left(Y_v^h + \tilde{Y}_v^h\right)\right)$.

Resistant, vaccinated, HLA+	$\frac{dR^1}{dt} = Bp\gamma r - \mu R^1$	(1)
Resistant, vaccinated, HLA-	$\frac{dR^0}{dt} = B(1-p)\gamma r - \mu R^0$	(2)
Susceptible, unvaccinated, HLA+	$\frac{dX^1}{dt} = Bp(1-\gamma) - (\lambda_0 + \lambda_1 + \mu)X^1$	(3)
Susceptible, unvaccinated, HLA-	$\frac{dX^0}{dt} = B(1-p)(1-\gamma) - (\lambda_0 + \lambda_1 + \mu)X^0$	(4)
Susceptible, vaccinated, HLA+	$\frac{d\tilde{X}^1}{dt} = Bp\gamma(1-r) - (\lambda_0 + \lambda_1 + \mu)\tilde{X}^1$	(5)
Susceptible, vaccinated, HLA-	$\frac{d\tilde{X}^0}{dt} = B(1-p)\gamma(1-r) - (\lambda_0 + \lambda_1 + \mu)\tilde{X}^0$	(6)
Infected (WT), unvaccinated, HLA+	$\frac{dY_0^1}{dt} = \lambda_0 X^1 - \phi_1 Y_0^1 - (\mu + \alpha)Y_0^1$	(7)
Infected (escape), unvaccinated, HLA+	$\frac{dY_1^1}{dt} = \lambda_1 X^1 + \phi_1 Y_0^1 - (\mu + \alpha)Y_1^1$	(8)
Infected (WT), unvaccinated, HLA-	$\frac{dY_0^0}{dt} = \lambda_0 X^0 + \psi_1 Y_1^0 - (\mu + \alpha)Y_0^0$	(9)
Infected (escape), unvaccinated, HLA-	$\frac{dY_1^0}{dt} = \lambda_1 X^0 - \psi_1 Y_1^0 - (\mu + \alpha)Y_1^0$	(10)
Infected (WT), vaccinated, HLA+	$\frac{d\tilde{Y}_0^1}{dt} = \lambda_0 \tilde{X}^1 - \tilde{\phi}_1 \tilde{Y}_0^1 - (\mu + \tilde{\alpha})\tilde{Y}_0^1$	(11)
Infected (escape), vaccinated, HLA+	$\frac{d\tilde{Y}_1^1}{dt} = \lambda_1 \tilde{X}^1 + \tilde{\phi}_1 \tilde{Y}_0^1 - (\mu + \alpha)\tilde{Y}_1^1$	(12)
Infected (WT), vaccinated, HLA-	$\frac{d\tilde{Y}_0^0}{dt} = \lambda_0 \tilde{X}^0 + \psi_1 \tilde{Y}_1^0 - (\mu + \alpha)\tilde{Y}_0^0$	(13)
Infected (escape), vaccinated, HLA-	$\frac{d\tilde{Y}_1^0}{dt} = \lambda_1 \tilde{X}^0 - \psi_1 \tilde{Y}_1^0 - (\mu + \alpha)\tilde{Y}_1^0$	(14)
