# Supplementary material for: Modelling the Spread of HIV Immune Escape Mutants in a Vaccinated Population
Source: PLoS Comput Biol. 2011 Dec 1;7(12):e1002289. doi: 10.1371/journal.pcbi.1002289 (PMC3228780; doi:10.1371/journal.pcbi.1002289)
Supplement: Text S3 — The next generation matrix for the single epitope version of the model. (PDF) [file pcbi.1002289.s006.pdf]

Text S3. The next generation matrix for the single epitope version of the model.

The control reproductive number (R_C) for the single epitope version of the vaccine model can be found by calculating the largest eigenvalue of this next generation matrix (**K**). The elements of the matrix, k_{lm}, define the expected number of new cases with state-at-infection *l*, generated by one individual who has just been born (epidemiologically speaking) in state-at-infection *m*. For the single epitope version of the model there are eight infectious states, however the model can be collapsed into a minimum of five states. Y_1^1 and $\tilde{Y}_1^1$ can be combined into a single state $\bar{Y}_1^1$ ($\bar{Y}_1^1 = Y_1^1 + \tilde{Y}_1^1$), which represents infected HLA-matched hosts with escape, and similarly for the pairs Y_0^0 and $\tilde{Y}_0^0$ ($\bar{Y}_0^0 = Y_0^0 + \tilde{Y}_0^0$), and Y_1^0 and $\tilde{Y}_1^0$ ($\bar{Y}_1^0 = Y_1^0 + \tilde{Y}_1^0$). The next generation matrix is therefore provided for the five states: $Y_0^1, \tilde{Y}_0^1, \bar{Y}_1^1, \bar{Y}_0^0, \bar{Y}_1^0$. The largest eigenvalue of this matrix is too complicated to provide analytically. However, we have used this matrix to numerically calculate the control reproductive number for different parameter sets and show how they affect this metric. Those findings are presented in Text S4.

$$
\mathbf{K} = \begin{pmatrix}
\frac{\beta c p(1-\gamma)}{\phi_1+\mu+\alpha} & \frac{\tilde{\beta}\tilde{c}p(1-\gamma)}{\tilde{\phi}_1+\mu+\tilde{\alpha}} & 0 & \frac{\beta c p(1-\gamma)}{\mu+\alpha} & \frac{\psi_1}{\psi_1+\mu+\alpha}\frac{\beta c p(1-\gamma)}{\mu+\alpha} \\
\frac{\beta c p\gamma(1-r)}{\phi_1+\mu+\alpha} & \frac{\tilde{\beta}\tilde{c}p\gamma(1-r)}{\tilde{\phi}_1+\mu+\tilde{\alpha}} & 0 & \frac{\beta c p\gamma(1-r)}{\mu+\alpha} & \frac{\psi_1}{\psi_1+\mu+\alpha}\frac{\beta c p\gamma(1-r)}{\mu+\alpha} \\
\frac{\phi_1}{\phi_1+\mu+\alpha}\frac{\beta c p(1-\gamma r)}{\mu+\alpha} & \frac{\tilde{\phi}_1}{\tilde{\phi}_1+\mu+\tilde{\alpha}}\frac{\beta c p(1-\gamma r)}{\mu+\alpha} & \frac{\beta c p(1-\gamma r)}{\mu+\alpha} & 0 & \frac{\beta c p(1-\gamma r)}{\psi_1+\mu+\alpha} \\
\frac{\beta c(1-p)(1-\gamma r)}{\phi_1+\mu+\alpha} & \frac{\tilde{\beta}\tilde{c}(1-p)(1-\gamma r)}{\tilde{\phi}_1+\mu+\tilde{\alpha}} & 0 & \frac{\beta c(1-p)(1-\gamma r)}{\mu+\alpha} & \frac{\psi_1}{\psi_1+\mu+\alpha}\frac{\beta c(1-p)(1-\gamma r)}{\mu+\alpha} \\
\frac{\phi_1}{\phi_1+\mu+\alpha}\frac{\beta c(1-p)(1-\gamma r)}{\mu+\alpha} & \frac{\tilde{\phi}_1}{\tilde{\phi}_1+\mu+\tilde{\alpha}}\frac{\beta c(1-p)(1-\gamma r)}{\mu+\alpha} & \frac{\beta c(1-p)(1-\gamma r)}{\mu+\alpha} & 0 & \frac{\beta c(1-p)(1-\gamma r)}{\psi_1+\mu+\alpha}
\end{pmatrix}
$$
