# Supplementary material for: Modelling the Spread of HIV Immune Escape Mutants in a Vaccinated Population
Source: PLoS Comput Biol. 2011 Dec 1;7(12):e1002289. doi: 10.1371/journal.pcbi.1002289 (PMC3228780; doi:10.1371/journal.pcbi.1002289)
Supplement: Text S4 — The impact that different parameters have upon the control reproductive number, RC, for the single epitope version of the vaccine (n = 1). (PDF) [file pcbi.1002289.s007.pdf]

Text S4. The impact that different parameters have upon the control reproductive number, R_C, for the single epitope version of the vaccine (n=1).

The first row of the table below provides an example set of parameters for the model and the resulting estimate of R_C, calculated using the next generation matrix (Text S3). In each of the remaining rows we increase only one of the parameters compared to the first example (the increase in each example is represented by an upwards pointing arrows in column 2). The effect that these parameter changes have on R_C is provided in column three. The arrows represent whether R_C increases (↑), decreases (↓) or remains invariant to the parameter change (↔). The figure below shows how R_C changes with HLA prevalence.

	Model parameters	R_C
An example set of parameters and corresponding value of R_C	$\gamma = 0.8$, $\phi_1 = 1/30$, $\tilde{\phi}_1 = 1/30$, $\psi_1 = 1/30$, $r = 0$, $\beta c = 0.2$, $\tilde{\beta} c = 0.04$, $1/(\mu+\alpha) = 10$, $1/(\mu+\tilde{\alpha}) = 30$, $1/\mu = 50$, $p^1 = 0.2$	1.911

Examples showing how changing one of the parameters from the set above would affect R_C<br><br>The arrows show whether an increase or decrease in the model parameter corresponds to an increase or decrease in R_C compared to R_C =1.911	Proportion vaccinated	$\gamma = 1$ (↑)	1.895 (↓)
	Rate of escape in unvaccinated hosts⁺	$\phi_1 = 1/10$ (↑)	1.914 (↑)
	Rate of escape in vaccinated hosts⁺	$\tilde{\phi}_1 = 1/10$ (↑)	1.959 (↑)
	Rate of reversion	$\psi_1 = 1/10$ (↑)	1.888 (↓)
	Proportion of vaccinated host resistant	$r = 0.3$ (↑)	1.456 (↓)
	Transmission coefficient of unvaccinated hosts	$\beta c = 0.4$ (↑)	3.811 (↑)
	Transmission coefficient of successfully vaccinated hosts	$\tilde{\beta} c = 0.2$ (↑)	2.251 (↑)
	Life expectancy of unvaccinated hosts	$1/(\mu+\alpha) = 20$ (↑)	3.779 (↑)
	Life expectancy of successfully vaccinated hosts	$1/(\mu+\tilde{\alpha}) = 50$ (↑)	1.942 (↑)
	Life expectancy of uninfected hosts*	$1/\mu = 70$ (↑)	1.911 (↔)

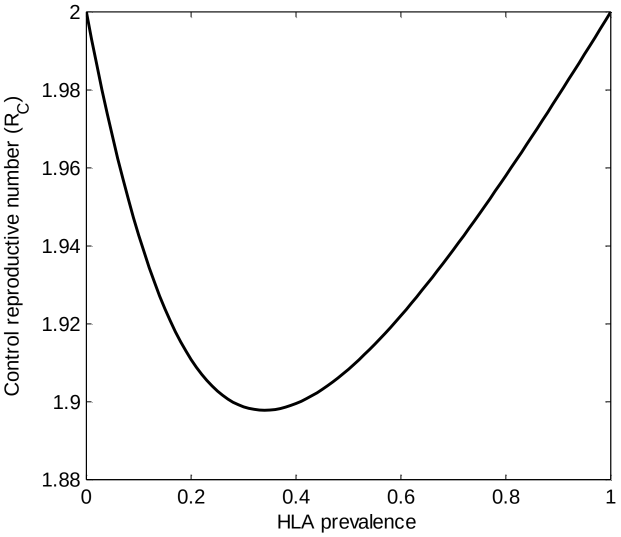

This analysis shows that R_C is larger when:

- the rate of escape in unvaccinated HLA-matched hosts (ϕ_1) is faster
- the rate of escape in vaccinated HLA-matched hosts ($\tilde{\phi}_1$) is faster
- the rate of reversion in HLA mismatched hosts (ψ_1) is slower
- the proportion of vaccinated hosts resistant to infection (r) is smaller
- the proportion of hosts who are vaccinated (γ) is smaller
- the transmission coefficient of unvaccinated and unsuccessfully vaccinated hosts (βc) is higher.
- the transmission coefficient of successfully vaccinated hosts ($\tilde{\beta} c$) is higher.
- the life expectancy of unvaccinated and unsuccessfully vaccinated hosts ($1/(\mu+\alpha)$) is longer.
- the life expectancy of successfully vaccinated hosts $(1/(\mu+\tilde{\alpha}))$ is longer.

Furthermore:

- R_C is invariant* to the life expectancy of uninfected hosts $1/\mu$.
- R_C changes according to a u-shaped function of HLA-prevalence (p^1), i.e. is lowest at an intermediate HLA prevalence.

*For consistency with standard notation, death rates of infected hosts ($\mu+\alpha$ for vaccinated hosts and $\mu+\tilde{\alpha}$ for unvaccinated hosts) are denoted as the sum of the death rate of uninfected hosts (μ) and an additional term (α and $\tilde{\alpha}$, respectively). However, in assessing how R_C changes with the life expectancy of uninfected hosts, $1/\mu$, we keep the life expectancy of vaccinated and unvaccinated hosts fixed.

+For this analysis we assume that the rate of escape in vaccinated ($\tilde{\phi}_1$) and unvaccinated (ϕ_1) hosts vary independently.
